# Shifts in fungal communities drive soil profile nutrient cycling during grassland restoration

**DOI:** 10.1128/mbio.02834-24

**Published:** 2025-01-24

**Authors:** Yuting Xu, Ke Cui, Xiaoshan Zhang, Guodong Diwu, Yuanjun Zhu, Lei Deng, Yangquanwei Zhong, Weiming Yan

**Affiliations:** 1 State Key Laboratory of Soil Erosion and Dryland Farming on the Loess Plateau, Northwest A & F University, Yangling, Shaanxi, China; 2 Shaanxi Key Laboratory of Qinling Ecological Intelligent Monitoring and Protection, School of Ecology and Environment, Northwestern Polytechnical University, Xi'an, China; 3 Institute of Soil and Water Conservation, Chinese Academy of Sciences, Yangling, Shaanxi, China; 4 Research & Development Institute of Northwestern Polytechnical University, Shenzhen, China; University of Tennessee at Knoxville, Knoxville, Tennessee, USA

**Keywords:** soil microbe, functional gene, life strategy, subsoil, grassland restoration

## Abstract

Soil microbial diversity and community life strategies are crucial for nutrient cycling during vegetation restoration. Although the changes in topsoil microbial communities during restoration have been extensively studied, the structure, life strategies, and function of microbial communities in the subsoil remain poorly understood, especially regarding their role in nutrient cycling during vegetation restoration. In this study, we conducted a comprehensive investigation of the changes in the soil microbial community, assembly process, life strategies, and nutrient cycling functional genes in soil profiles (0–100 cm) across a 36 year chronosequence (5, 15, 28, and 36 years) of fenced grassland and one grazing grassland on the Loess Plateau of China. Our results revealed that soil organic carbon increased by 76.0% in topsoil and 91.6% in subsoil after 36 years of restoration. The bacterial communities were influenced primarily by soil depth, while the fungal communities were highly sensitive to the years of restoration. Microbes in the subsoil recovered faster, and the microbial community structure and functional genes in the soil profiles gradually became more consistent following restoration. In addition, we observed a transition in microbial life history strategies from a persistent *K*-strategy to a rapid *r*-strategy during restoration. Notably, the fungal community assembly process played an important role in changes in nutrient cycling genes, which were accompanied by increased carbon fixation and nitrogen mineralization function. Overall, our findings provide several novel insights into the impact of changes in the fungal community on soil nutrient cycling in the soil profile during vegetation restoration.

**IMPORTANCE:**

Our study revealed that microbes in the subsoil recovered faster than those in the topsoil, which contributed to the reduction in differences in microbial community structure and the distribution of functional genes throughout the soil profile during the restoration process. Importantly, the assembly of fungal communities plays a pivotal role in driving changes in nutrient cycling genes, such as increased carbon fixation and nitrogen mineralization, alongside a reduction in carbon degradation gene abundance. These alterations increase soil organic carbon and nutrient availability during restoration. Our results increase the understanding of the critical role of fungal communities in soil nutrient cycling genes, which facilitate nutrient accumulation in soil profiles during grassland restoration. This insight can guide the development of strategies for manipulating fungal communities to increase soil nutrients in grasslands.

## INTRODUCTION

Grazing exclusion, as a “nature-based” ecoengineering measure, is used globally to restore degraded grassland ecosystems (1, 2). Soil microbes play crucial roles in ecological processes, including decomposing soil organic carbon (SOC) (3), facilitating soil nutrient cycling (4), and promoting plant growth (5), which are recognized as key factors driving vegetation restoration (6, 7). While soil microbes in topsoil have been extensively studied in the context of vegetation restoration, there is a limited understanding of whether, how, and why the soil microbial community and its functions in the subsoil vary throughout this process. Investigating dynamic changes in the soil microbial community and its functions in the soil profile could enhance our knowledge of the vertical turnover of soil microbes following grassland restoration.

Vegetation restoration impacts the soil microbe community and function through both direct and indirect effects on biotic and abiotic factors (8, 9). In general, vegetation restoration increases above- and belowground biomass (6, 10), thereby increasing SOC inputs and nutrient availability (11). Driven by changes in soil substrates and plant communities following vegetation restoration, soil microbial community composition and life strategies can adjust simultaneously with environmental changes (12–14). Moreover, the corresponding functions of soil microbes also shift, such as the decline in C degradation gene abundances along with long-term restoration, due to changes in litter characteristics and SOC (7, 15, 16). In general, bacteria excel in terms of environmental adaptability and the acquisition of nutrient sources (17), whereas fungi are superior in terms of enzyme degradation of SOC (18, 19); thus, bacterial and fungal communities exhibit different responses to restoration (20). However, the relative roles of bacterial and fungal communities in soil nutrient cycling during grassland restoration remain to be revealed.

Previous studies have addressed subsoils as entities that are distinct from the surface due to different environmental peculiarities (e.g., inputs of fresh SOC, soil aeration, and substrate availability) (21, 22). Microorganisms in topsoil utilize C sources more easily than those in subsoil because of the greater input of C from litter and root exudates, which results in a faster C decomposition rate (23). The subsoil is far more substrate limited, as less available substrate is delivered from plants (23, 24). Thus, microbial biomass often decreases sharply with increasing soil depth, but the effects of soil microbes on biogeochemical processes persist throughout the soil profile (25). Following grassland restoration, plants progressively develop deeper and more complicated root systems with the replacement of plant species (26, 27), impacting the input of fresh soil C and substrate availability in the soil profile (28). These substrates provide primary C and energy sources for subsoil microorganisms and increase the growth and evolution of these microbes (29, 30). This further impacts the functions and structure of soil microbial communities (26, 31, 32); however, it is unclear whether the microbial communities in the soil profile respond consistently to vegetation restoration. Therefore, elucidating the variations in the soil microbial community and functions in the soil profile could increase our understanding of the role that microbes play in the subsoil in SOC and nutrient accumulation during the vegetation restoration.

A comprehensive understanding of the changes in soil microbial communities and functions in the soil profile following grassland restoration can help reveal the role of bacteria and fungi in promoting soil nutrient cycling. In this study, we investigated the changes in bacterial and fungal communities and nutrient cycling gene abundance in one grazed grassland and four grasslands with different fenced years (5, 15, 28, and 36 years). We hypothesize that (i) bacterial and fungal communities in the soil profile respond differently to grassland restoration; (ii) changes in the assembly processes of bacterial and fungal communities are driven differently by restoration years and soil physicochemical properties; and (iii) fungal communities might play a more important role in changes in nutrient cycling functional genes during grassland restoration.

## RESULTS

### Soil physicochemical properties and microbial community changes in the soil profile during vegetation restoration

The vegetation coverage increased from 64.7% to 90.3% after 36 years of grazing exclusion (Fig. S1), and the SOC increased by 76.0% in the topsoil (0–40 cm) and by 91.6% in the subsoil (40–100 cm). In addition, the total nitrogen (TN) content also increased from 1.69 to 2.92 g kg^−1^ in the topsoil and from 0.96 to 1.81 g kg^−1^ in the subsoil (Tables S1 and S2). Compared with those of the topsoil, the soil physicochemical properties of the subsoil showed more pronounced variations (*P* < 0.01) across restoration years (Fig. S2A), and the differences in the soil physicochemical properties between the topsoil and the subsoil decreased as the vegetation restoration progressed (Fig. S2B).

**Fig 1:**
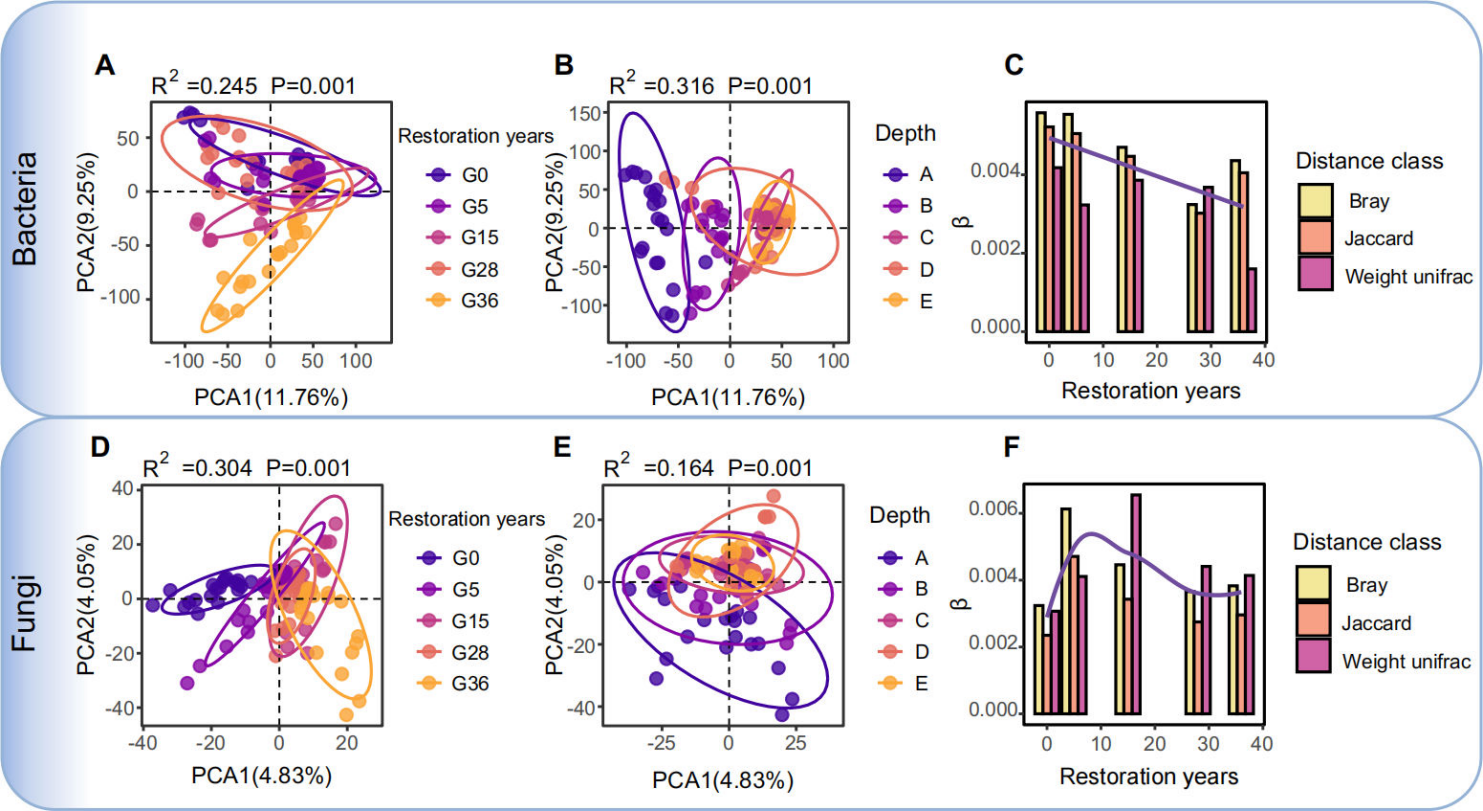
Bacterial and fungal community diversity and spatial turnover rate. PCA of the bacterial community based on the Bray–Curtis distance separated by restoration years (**A**) and soil depth (**B**) (*P* < 0.001, PERMANOVA by Adonis). PCA analysis of the fungal community based on Bray–Curtis distances separated by restoration year (**D**) and soil depth (**E**). The ellipses cover 95% of the data for each group. The spatial turnover rates of the bacterial (**C**) and fungal (**F**) communities were determined on the basis of the Bray–Curtis, Jaccard, and weighted UniFrac distances. A, 0–20 cm; B, 20–40 cm; C, 40–60 cm; D, 60–80 cm; E, 80–100 cm.

Soil depth and restoration years strongly impacted both the bacterial and fungal communities. The bacterial community appeared to be more strongly influenced by soil depth (*R*^2^ = 0.316, *P* < 0.001) than restoration year (*R*^2^ = 0.245, *P* < 0.001), whereas the fungal community was more sensitive to restoration year (*R*^2^ = 0.304, *P* < 0.001) than to soil depth (*R*^2^ = 0.164, *P* < 0.001) (Fig. 1; Table S3). The alpha diversity of the bacterial and fungal communities decreased as the soil depth increased (Fig. S3). Interestingly, the differences in the microbial community spatial turnover rates and compositions among the different soil depths decreased with increasing restoration years (Fig. 1; Fig. S4; Table S4), which exhibited varying correlations with the soil physicochemical properties (Table S5).

**Fig 2:**
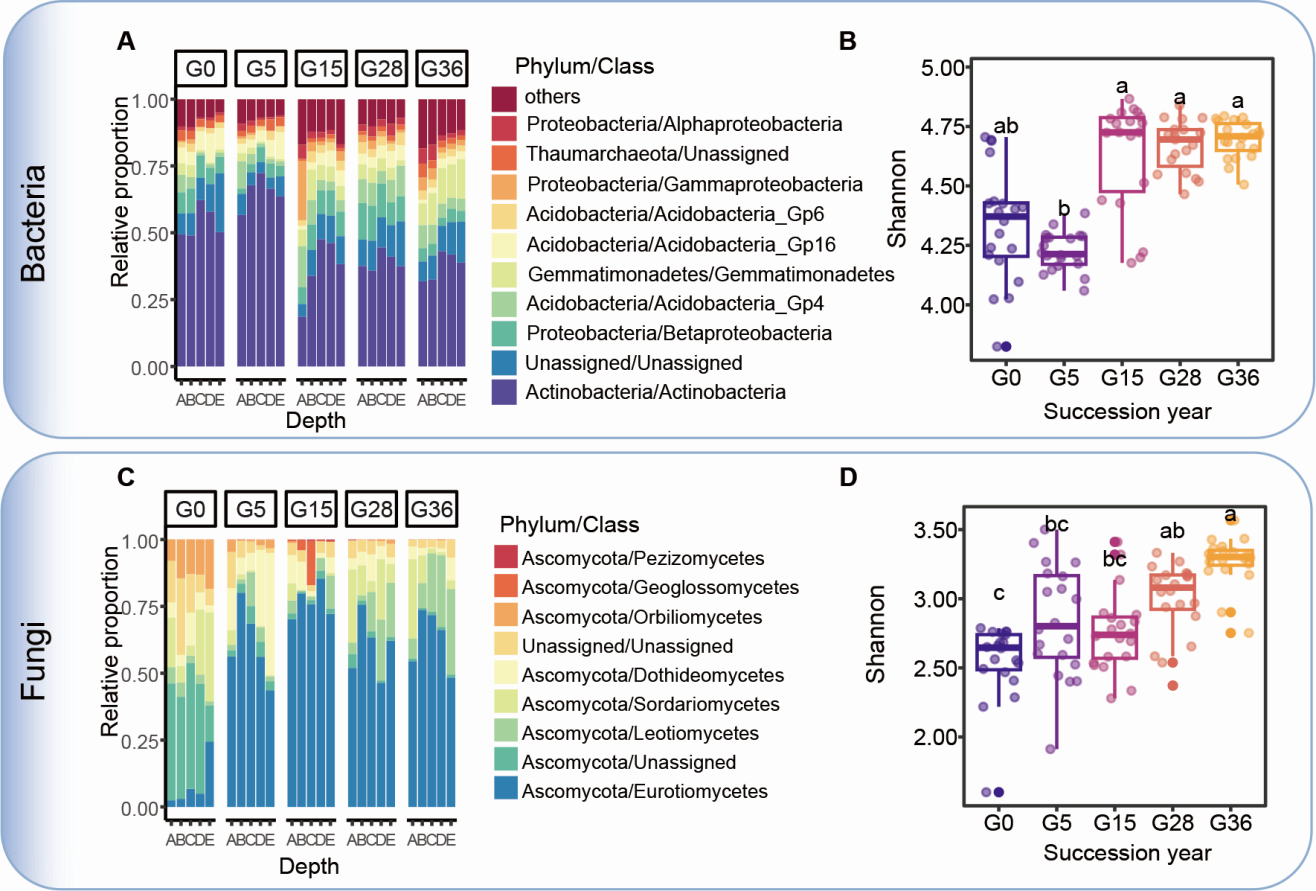
Restoration-discriminant microbial species composition at the class level and Shannon diversity in the soil profile following restoration. Lowercase letters indicate significant differences among restoration years, as determined by one-way ANOVA and Duncan’s multiple-range test. A, 0–20 cm; B, 20–40 cm; C, 40–60 cm; D, 60–80 cm; E, 80–100 cm.

A total of 73% bacterial and 36% fungal amplicon sequence variants (ASVs) were detected in all the soil samples during vegetation restoration (Fig. S5). Thus, we identified the important ASVs that correlated with restoration year using a random forest model with 10-fold cross-validation, and 397 bacterial species and 131 fungal species were distinguished as biomarker taxa in the model in order of the degree of discriminatory importance of the restoration year (Tables S6 and S7). The Shannon index of the restoration-discriminant bacteria was lower in the early stage (0 to 5 years) and then reached a steady value at the later stage (15 to 36 years), whereas the Shannon index of the restoration-discriminant fungi gradually increased from the early to the late restoration stage (Fig. 2).

### Assembly process and life strategy shifts of the microbial community in the soil profile during restoration

**Fig 3:**
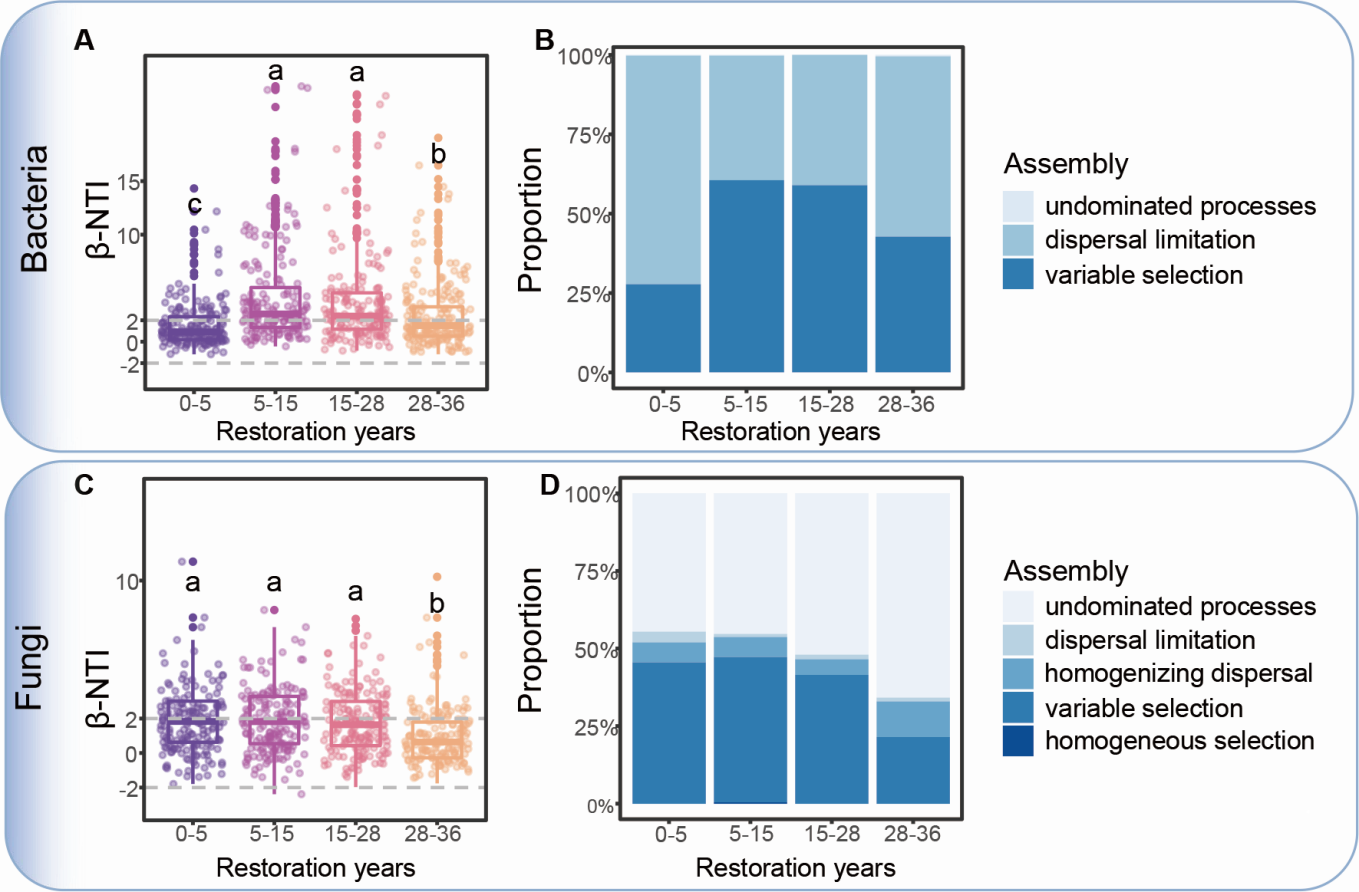
Bacterial community assembly process during the restoration stage. (**A**) βNTI in different bacterial communities. (**B**) Bacterial community assembly process classification. (**C**) βNTI in different fungal communities. The horizontal dashed gray lines indicate upper and lower significance thresholds at |βNTI| < 2. (**D**) Classification of fungal community assembly processes. To better express the changes in assembly machinery during restoration, we calculated the assembly process between the adjacent two restoration stages: 0 to 5 years, 5 to 15 years, 15 to 28 years, and 28 to 36 years. Lowercase letters indicate significant differences among restoration years, as determined by one-way ANOVA and Duncan’s multiple-range test.

The bacterial assembly processes were driven primarily by dispersal limitation at 0–5 years (72.0%), whereas the variable selection increased to 58.4% at 5–15 years and 55.4% at 15–25 years (Fig. 3A and B; Table S8). In contrast, fungal assembly processes were dominated by variable selection at 0–5 years (45.5%) and 5–15 years (46.8%), and then shifted to undominated processes at 25–36 years (65.8%) (Fig. 3C and D; Table S8). Moreover, the deterministic assembly process of the bacterial and fungal communities decreased from 0 to 80 cm and then increased in the soil at 80–100 cm (Fig. S6).

**Fig 4:**
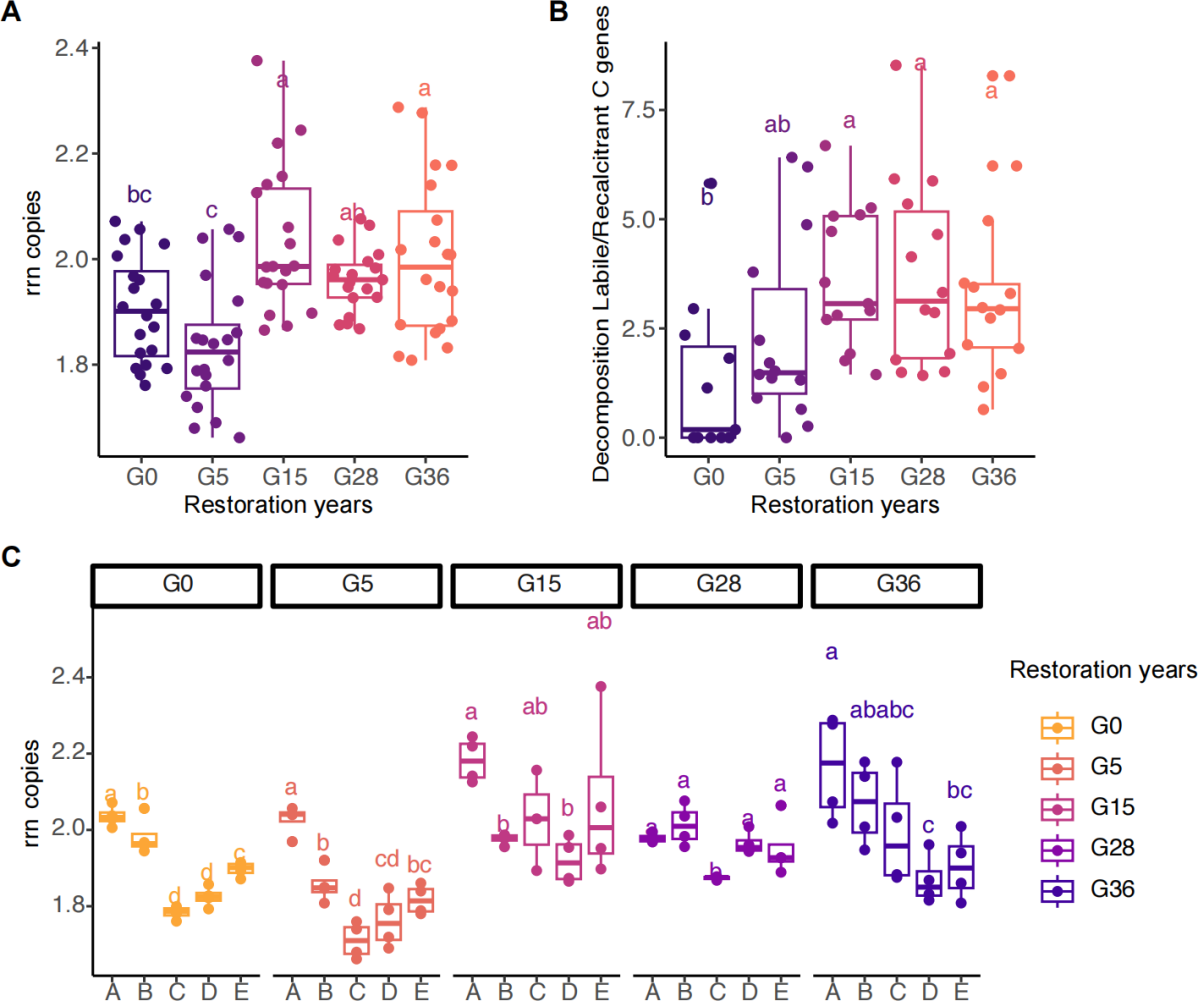
Life strategies of the soil microbial community in the soil profile following the restoration. (**A**) rRNA operon (rrn) copy number of bacteria; (**B**) labile to recalcitrant carbon degradation gene ratio; and (**C**) rRNA operon (rrn) copy number among different soil layers during restoration. Lowercase letters indicate significant differences among restoration years, determined by one-way ANOVA and Duncan’s multiple-range test. A, 0–20 cm; B, 20–40 cm; C, 40–60 cm; D, 60–80 cm; E, 80–100 cm.

The ribosomal RNA gene operon (rrn) copy number was used to distinguish the ecological strategies of bacterial communities, and a relatively high rrn copy number in bacteria is typically a feature of eutrophic bacteria (*r*-strategy), whereas a relatively low rrn copy number can be a characteristic of oligotrophic bacteria (*K*-strategy). The rrn copy numbers initially decreased from G0 to G5 but subsequently increased to G36 (Fig. 4A). Interestingly, the rrn copy numbers decreased and tended to favor the *K*-strategy in the subsoil (Fig. 4C). Furthermore, the ratio of labile (starch and hemicellulose) to recalcitrant carbon functional gene abundance (cellulose hydrolysis, chitin hydrolysis, pectin hydrolysis, and lignin hydrolysis) also exhibited the same trend as the rrn copy numbers of the microbial communities (Fig. 4B).

### Nutrient cycling functional gene abundance changes in the soil profile during restoration

**Fig 5:**
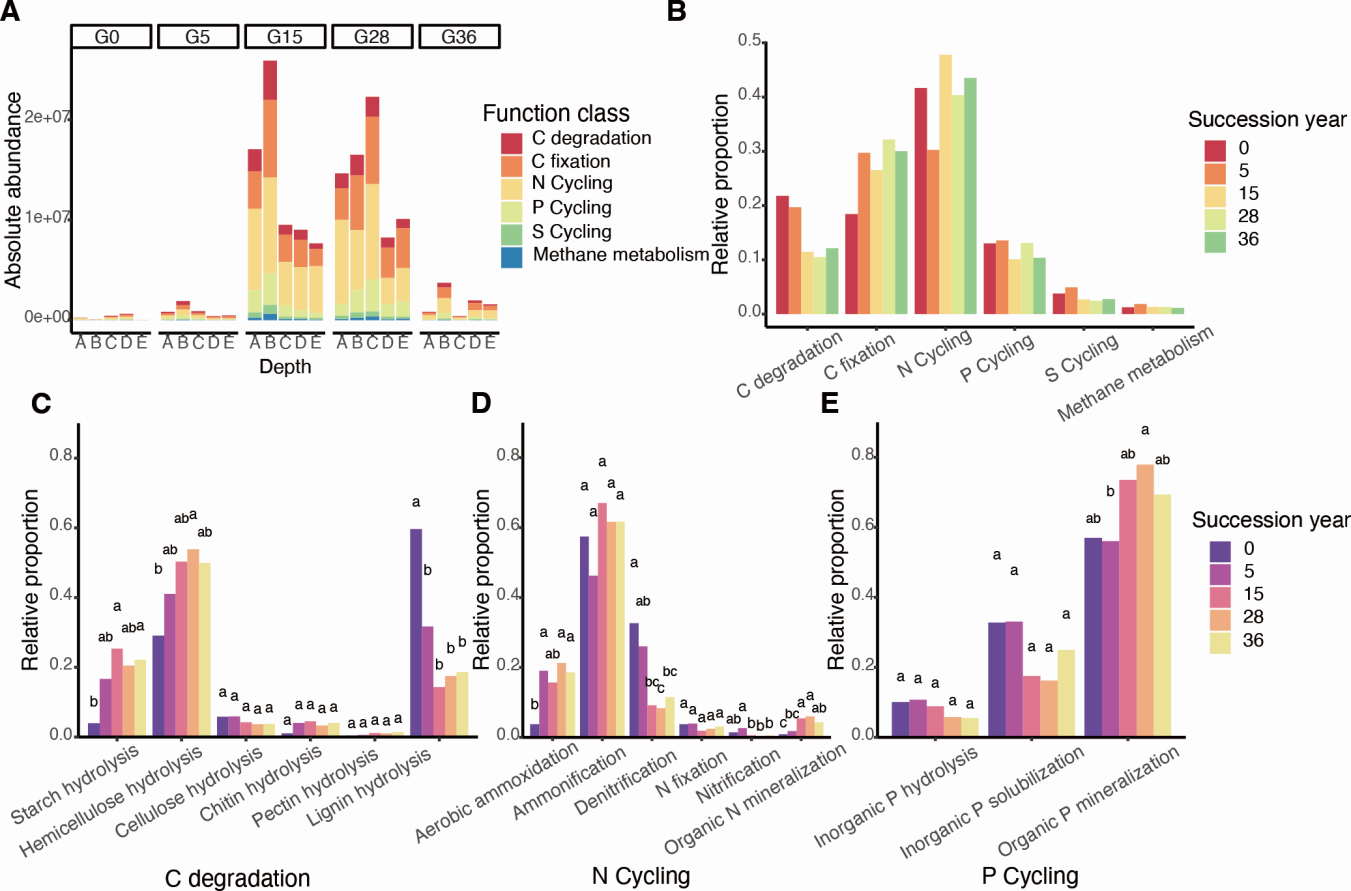
Changes in the abundance of microbial nutrient cycling functional genes during restoration. Absolute (**A**) and relative (**B**) abundances of soil microbial functional gene classes during restoration. Relative abundances of (**C**) carbon degradation, (**D**) nitrogen cycling, and (**E**) phosphorus cycling functional genes during restoration. Lowercase letters indicate significant differences among restoration years, as determined by one-way ANOVA and Duncan’s multiple-range test.

In the study, a total of 71 microbial functional genes related to C, N, P, and S cycling were detected via qPCR. The absolute abundance of functional genes first increased, and peaked at G15 and G28, and then decreased as the restoration progressed (Fig. 5A), which was consistent with the change in bacterial biomass (Fig. S7). The diversity and composition of nutrient cycling genes significantly shifted with the restoration year, and the fungal diversity (Chao1 index) gradually increased with the restoration year (Fig. S8; Table S9). Among all types of nutrient cycling functional genes, there was a distinct increase in the C fixation function (from 18.4% to 30.1%) and a decrease in the C degradation function (from 21.8% to 12.1%) from G0 to G36 (Fig. 5B). The C degradation functions of starch and hemicellulose hydrolysis increased from 3.1% in G0 to 22.2% in G36, and 22.9% in G0 to 45.0% in G36. The lignin hydrolysis gene abundance decreased from 68.3% in G0 to 18.6% in G36 (Fig. 5C). Moreover, aerobic ammoxidation genes and organic N mineralization genes increased, whereas denitrification genes decreased (Fig. 5D) and organic P mineralization in the P cycle increased following restoration (Fig. 5E). In addition, the discrepancies in microbial functions between topsoil and subsoil gradually diminished during restoration (Fig. S9).

### Different responses of the microbial community and functional gene abundance in topsoil and subsoil to restoration

**Fig 6:**
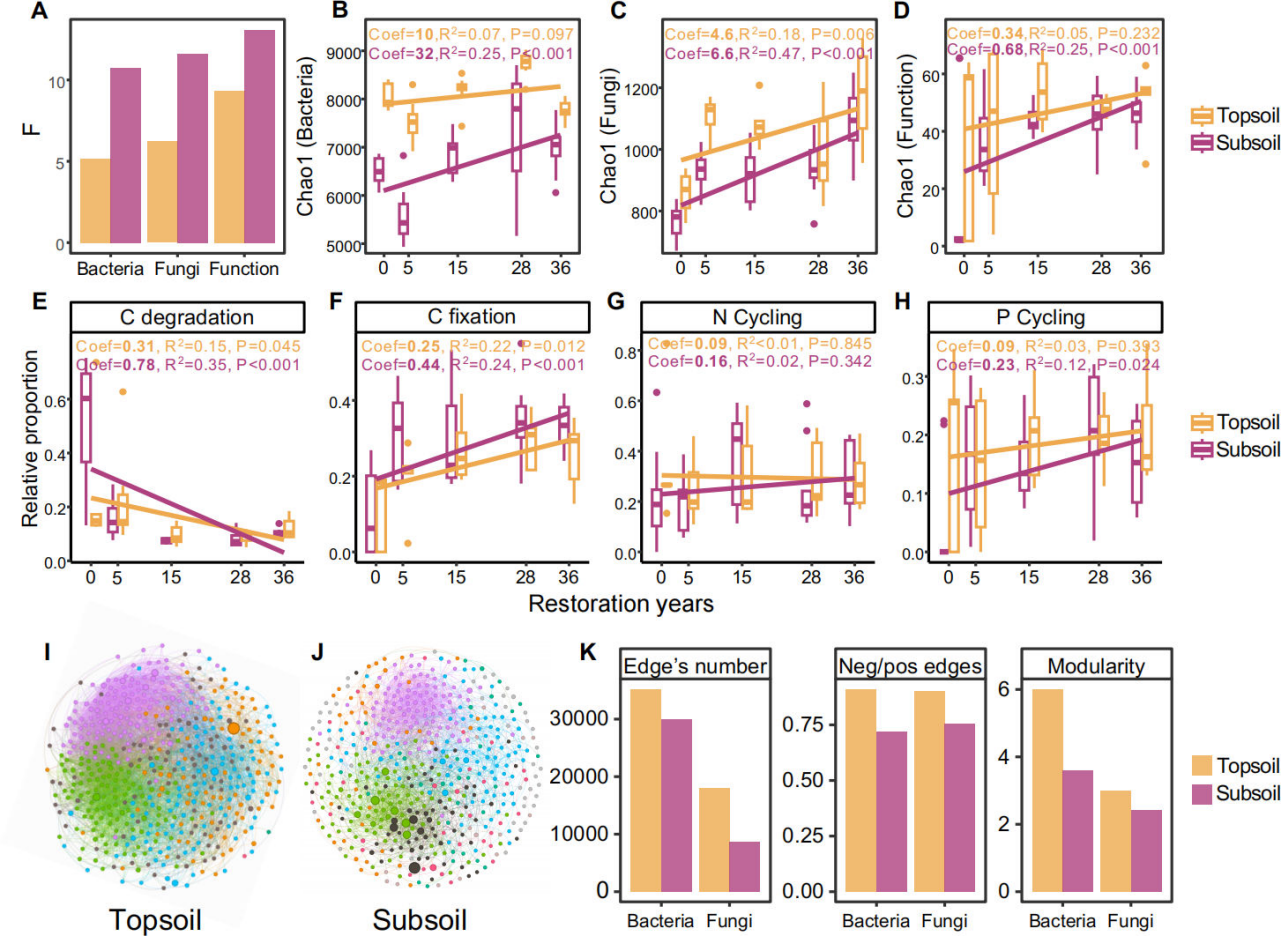
Differences in microbial diversity, nutrient cycling functional genes, and co-occurrence network structure between the topsoil (0–40 cm) and subsoil (40–100 cm) during restoration. (**A**) The microbial structure and nutrient cycling functional genes in the topsoil and subsoil were analyzed using a variance test based on the Bray–Curtis distance, and a higher F value indicates a greater difference. Linear fitting between bacterial (**B**), fungal (**C**), and nutrient cycling (**D**) gene diversity and the restoration year of topsoil and subsoil. Linear fitting between the relative abundances of C degradation (**E**), C fixation (**F**), N cycling (**G**), and P (**H**) cycling genes and the restoration year in the topsoil and subsoil. Coef, the magnitude of the coefficient for the linear term. Fungal co-occurrence network in the topsoil (**I**) and subsoil (**J**) during restoration. (**K**) Number of edges, ratio of negative to positive edges, and modularity of the bacterial and fungal co-occurrence network.

Compared with those in the topsoil layer, the microbial communities and nutrient cycling functional genes in the subsoil were largely influenced by the restoration year (Fig. 6A). Similarly, the alpha diversity (Chao1) of the bacteria, fungi, and nutrient cycling genes in the subsoil layer responded more intensely to restoration than those in the topsoil (Fig. 6B through D). The relative proportion of nutrient cycling genes also showed more pronounced changes in the subsoil with restoration years (Fig. 6E through H; Fig. S10). However, the microbial co-occurrence networks were more complex in the topsoil than in the subsoil, and greater number of edges, negative/positive edge rates, and relative modularity of bacteria and fungi were observed in the topsoil (Fig. 6I through K). Moreover, the Mantel test results also revealed a stronger relationship between the bacterial and fungal communities and the soil physicochemical properties in the subsoil (Table S10).

### Driving mechanism of the changes in the soil microbial community structure and function during restoration

**Fig 7:**
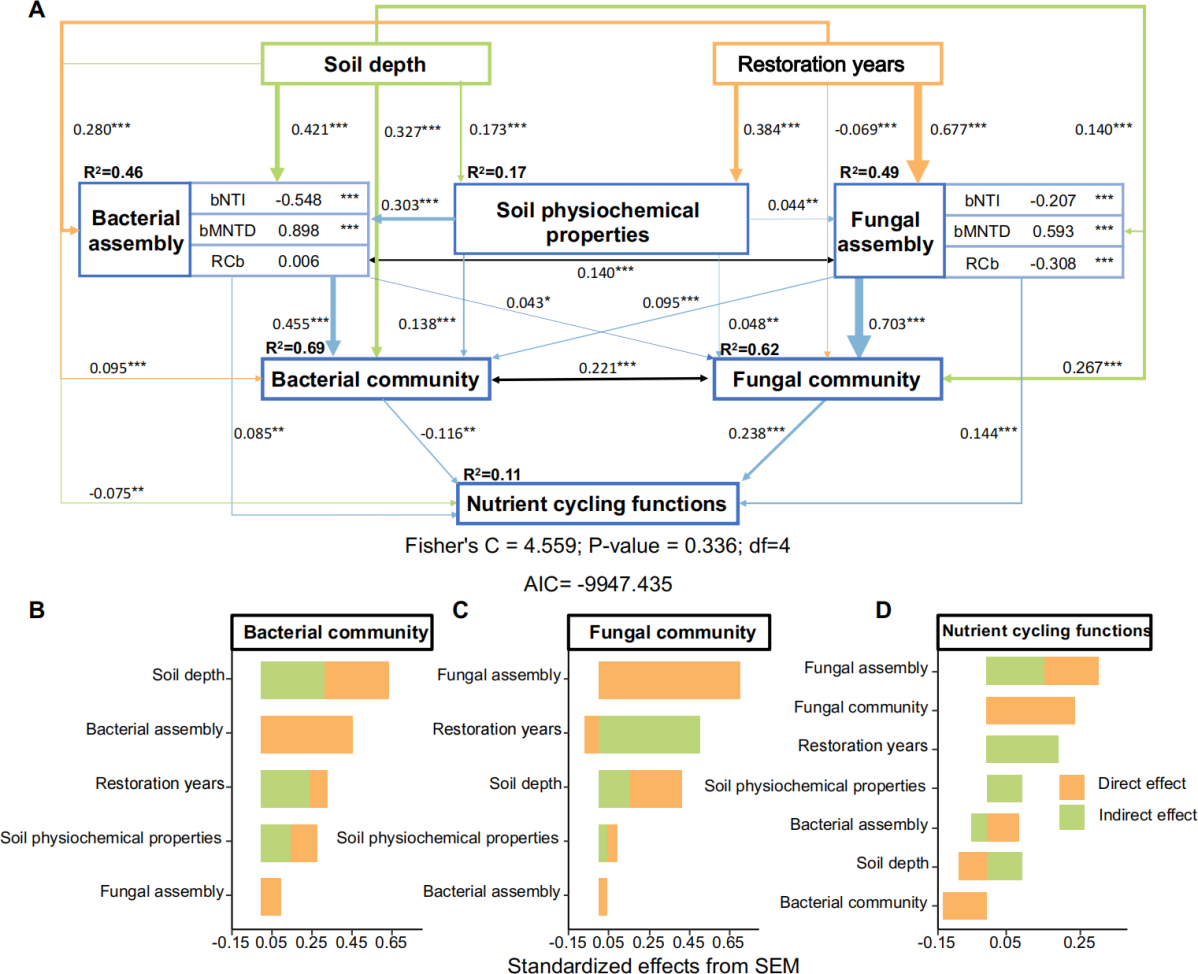
Structural equation model of the microbial community and functional changes during vegetation restoration. (**A**) Structural equation model and the direct and indirect effects of the composite variables on the bacterial community (**B**), fungal community (**C**), and nutrient cycling functional genes (**D**). The number of neighbors with the same direction as the arrow represents the path coefficient, and the width of the arrow is proportional to the saliency strength. *R*^2^ values represent the proportion of variance explained by each variable. The significance level is expressed as follows: *, *P* < 0.05; **, *P* < 0.01; ***, *P* < 0.001.

Structural equation modeling (SEM) was employed to gain further insight into the factors driving the changes in the soil microbial community structure and function during vegetation restoration (Fig. 7). The SEM revealed that soil depth (path coefficient = 0.67) plays a crucial role in shaping bacterial communities (*R*^2^ = 0.69) through both direct and indirect influences by altering soil physicochemical properties and the bacterial assembly process (Fig. 7A and B), whereas the fungal community (*R*^2^ = 0.62) is affected by both the restoration year (path coefficient = 0.44) and the soil depth (path coefficient = 0.42). The year of restoration influences the fungal community indirectly through its impact on fungal assembly, whereas soil depth directly influences the fungal community (Fig. 7C). SEM also revealed that nutrient cycling functional genes are driven primarily by changes in the fungal assembly process (path coefficient = 0.30), fungal communities (path coefficient = 0.24), and years of restoration (path coefficient = 0.19) (Fig. 7D), whereas shifts in the bacterial community negatively affect nutrient cycling.

## DISCUSSION

### Changes in edaphic factors drive the distinct responses of bacterial and fungal communities to grassland restoration

Our results indicate that the soil microbial community shifted in the 0–100 cm soil profile following grassland restoration, which was driven by soil nutrient availability in the soil profile during vegetation restoration (Fig. 1; Tables S1, S2, and S5) (8). However, we found that the fungal community was more sensitive to restoration than the bacterial community, which is consistent with previous studies (33, 34), possibly because fungi have stronger associations with plants (e.g., by serving as plant symbionts) and are largely influenced by changes in plants during restoration (7, 35). In addition, soil nutrients impact the response of microbes. We found that the bacterial communities were more strongly associated with the SOC and TN levels, which are more affected by soil depth than by restoration year (Tables S2 and S5). However, fungal communities presented a stronger correlation with NH_4_^+^, the primary nitrogen form utilized by fungi, which is less influenced by soil depth, indicating that fungi are highly dependent on soil nutrient availability (8, 36).

According to the size-plasticity hypothesis, smaller bacteria possess a broader ecological niche and exhibit a wider range of environmental adaptations than fungi (4, 37). During G0 to G5 , minor environmental changes had a weak impact on the bacterial community, as demonstrated by the weaker variable selection observed in this stage (Fig. 3A and B; Table S8); thus, bacteria did not significantly change during the 0 to 5 year natural restoration period (38). After the accumulation of nutrient resources from the G5 to G15, the bacterial community is strongly influenced by environmental changes, as demonstrated by the sudden increase in variable selection in stages G5 to G15 and G15 to G28. Conversely, even minor environmental changes can exert strong selection pressure on the fungal community, leading to sustained changes, as evidenced by the highly variable selection of the fungal assembly process from 0 to 28 years (Fig. 3C and D).

### Grassland restoration enhances microbial C fixation and reduces C degradation gene abundance

Increased nutrient availability also drives changes in soil microbial nutrient cycling gene abundance during restoration (7, 39). Our results revealed that grassland restoration enhances the microbial C fixation capacity, reduces the C degradation capacity, and increases functional alpha diversity (Fig. 5; Fig. S8) (7, 14). The greater C degradation ability in the early restoration stage was attributed mainly to the requirement of microbial growth, which needs to break down recalcitrant organic matter to acquire C because of the low C availability and fixation (7). As restoration progresses, the increased plant C input promotes the redistribution of microbial functions and energy, reducing their capacity for C degradation and enhancing their ability to acquire other nutrients to maintain their growth; thus, a greater abundance of N- and P-cycling genes was observed in the later restoration stage (Fig. 5A). The increase in soil nutrient availability also altered the life strategies of the microorganisms. Consequently, we observed an increase in the ratio of labile to recalcitrant C functional genes and rrn copy numbers during restoration (Fig. 4). These changes indicate that microbes shift from *K-*strategists to *r*-strategists and prefer to acquire easily degradable C (9, 14). Interestingly, the abundance of functional genes and bacterial biomass increased after the initial 28 years of restoration but sharply decreased at 36 years (Fig. 5A; Fig. S7), which could be attributed to a decreased plant community biomass and diversity (38, 40).

### Differences in the microbial community and function between topsoil and subsoil during grassland restoration

The results revealed that the microbial community diversity and function in subsoil were more sensitive to restoration than those in topsoil (Fig. 6; Fig. S2 and S10; Table S10). After grazing exclusion, the increased plant biomass increased the soil nutrient input in both the topsoil and the subsoil (Table S1) (6), subsequently triggering simultaneous changes in both microbial community and functions (30, 41), which further contributed to soil nutrient retention. The positive feedback loop between the microbial community and soil nutrients promotes the recovery of the grasslands during restoration (42). However, the low nutrient availability of the subsoil led to a stronger relationship between soil microbes and soil nutrients (Table S10) (43). Moreover, microbes in the subsoil are more inclined to cooperate rather than compete to maximize the utilization of limited resources and adapt to environmental pressures (Fig. 6K) (44). Compared with the topsoil, the stronger relationships and cooperation among microorganisms enhance the recovery of soil microbes in the subsoil during restoration. In addition, vegetation restoration facilitates an increase in soil nutrients in the subsoil (Fig. S2), which enhances the stability and consistency of the soil environment, leading to a reduced spatial turnover rate of the soil microorganisms, as well as a decrease in differences in microbial composition and functional genes among soil profiles over successive years (Fig. 1; Fig. S2 and S9).

### Nutrient cycling functional gene changes are driven mainly by the fungal community and related assembly processes

Nutrient cycling is predominantly influenced by the fungal assembly process, fungal community composition, and restoration year (Fig. 7). During restoration, the fungal assembly process experiences consistently high selection pressure due to changing environmental conditions, which are specifically related to resource availability. This ongoing selection pressure significantly influences the composition and structure of fungal communities. Fungi have an efficient extracellular enzyme system and a large mycelial network, providing them with an advantage in the decomposition of complex substrates (plant residues, cellulose, etc.) (16, 18, 19). Furthermore, fungal mycelia can form symbiotic relationships with plant roots, connecting plants and soil to establish an “expressway” for C transport. This allows fungi to actively participate in the input, stabilization, and decomposition of SOC (45). The powerful organic matter degradation and connectivity abilities of fungi position them as the main drivers of soil nutrient cycling, effectively promoting the nutrient release of plant residues and nutrient cycling in grassland ecosystems (46). In contrast, bacteria play a slightly inferior role in nutrient cycling. Bacterial DNA is in a free state within the cell, and an average of 42.5% of genes in the genome are affected by horizontal gene transfer (47),which allows bacteria to dynamically adjust their functions to adapt to new environmental conditions (48, 49) without largely altering their community composition. This poses a challenge in accurately assessing the influence of the bacterial community on functional genes. Moreover, the low *R*^2^ (0.11) for the nutrient cycling functional genes determined by the SEM indicates that the majority of the variability in the nutrient cycling function remains unexplained.

In this study, we investigated the changes in the soil microbial community and nutrient cycling functional genes across soil profiles following vegetation restoration. Our findings indicate that soil depth predominantly influences bacterial communities, whereas fungal communities are highly sensitive to the duration of restoration. Notably, microbes in the subsoil recovered faster than those in the topsoil, which contributed to a reduction in differences in microbial community structure and the distribution of functional genes throughout the soil profile during the restoration process. Importantly, the fungal community assembly process played a pivotal role in driving changes in nutrient cycling functional genes, such as increased carbon fixation and nitrogen mineralization, in addition to a reduction in the abundance of genes involved in carbon degradation. These alterations contributed to increased SOC and nutrient accumulation. Overall, our results increase the understanding of the critical role of fungal communities in influencing changes in soil nutrient cycling genes, thereby facilitating nutrient accumulation in soil profiles during grassland restoration.

## MATERIALS AND METHODS

### Study site and soil sampling

The study was conducted in the Yunwushan National Natural Grassland Protection Zone on the Loess Plateau (106°21′–106°27′E, 36°10′–36°17′N), China. The region experiences a semiarid climate, with a mean annual precipitation of 425 mm and an average annual temperature of 7°C. In August 2018, we selected a chronosequence of four restoration habitats fenced between 1984 and 2013, corresponding to 36 years (G36), 28 years (G28), 15 years (G15), and 5 years (G5) of restored grasslands, and a continuously grazed grassland was also included (G0).

Each habitat was >1 ha in size, and we selected a 100 m long strip for sampling and established four replicate plots (2 × 2 m quadrats) at 20 m intervals. Five soil cores (5 cm in diameter) were randomly collected from the soil profile (0–20, 20–40, 40–60, 60–80, and 80–100 cm, referred to as A, B, C, D, and E, respectively) in each plot and were thoroughly mixed to produce a composite soil sample. All soil samples were sieved through a 2 mm screen to remove roots and other debris. Each bulk sample was then divided into two subsamples. One subsample was promptly stored at −80°C to allow for DNA extraction, while the other subsample was air dried to facilitate physicochemical analysis.

### Analysis of soil physicochemical properties

The soil pH was measured using a pH meter after shaking the soil–water (1:2.5 [wt/vol]) suspension for 30 minutes. Air-dried soil passed through a 100 mesh sieve was used to measure the SOC content with dichromate oxidation, and the TN content using the Kjeldahl method (50). The soil samples were shaken in 50 mL of 1.0 mol l^−1^ KCl for 30 minutes to extract soil NH_4_^+^ and NO_3_^−^, and the concentrations of NH_4_^+^ and NO_3_^−^ were determined after filtration using a continuous flow analytical system (Autoanalyzer 3, Bran + Luebbe, Germany) (51) (Table S1).

### DNA extraction, polymerase chain reaction amplification, and data processing

Microbial DNA was extracted from a 0.5 g soil sample using an E.Z.N.A. soil DNA kit (Omega Biotek, Norcross, GA, USA). The V4 regions of the bacterial and archaeal 16S rRNA genes and ITS1 were amplified via polymerase chain reaction (PCR) using the Phusion High-Fidelity PCR Master Mix (New England Biolabs, New Ipswich, MA, USA). The PCR conditions consisted of an initial denaturation step at 95°C for 2 minutes, followed by 27 cycles of denaturation at 95°C for 30 seconds, annealing at 55°C for 30 seconds, extension at 72°C for 45 seconds, and a final extension step at 72°C for 10 minutes.

Reads generated from 16S V4 and ITS1 sequencing were analyzed using QIIME 2 (v.2018.4), and the DADA2 algorithm was employed to dereplicate the reads via paired-end settings (52). For the ITS1 reads, the same dereplication algorithm was employed with single-end settings. This resulted in ASV tables with read counts. The taxonomic assignment of the representative ASVs for the 16S V4 region was performed using the SILVA 128 database and the naïve Bayes classifier within QIIME 2 (53). Phylogenetic trees were constructed in QIIME 2 using the MAFFT alignment method coupled with the FastTree algorithm. The sequencing data for the archaeal and bacterial 16S rRNA genes and the fungal ITS gene were deposited into the NCBI SRA database under the accession number PRJNA1138184.

### Microbial nutrient cycling gene abundance detection by high-throughput qPCR

High-throughput qPCR was used to detect the functional genes of the soil microbial community related to carbon, nitrogen, phosphorus, and sulfur cycling in the microbial samples (54), and a detailed list of 71 genes is shown in Table S11. Microbial DNA extracted from the samples was tested for total amount and purity. Qualified DNA samples and qPCR reagents were added to a 384-well plate as a sample source plate, and primers and qPCR reagents were added to another 384-well plate as an assay source plate. A SmartChip Multisample Nanodispenser was used to add the sample source plate and assay source plate reagents to the micropores of the high-throughput qPCR SmartChip MyDesign Chip (Takara Biomedical Technology, Clontech). qPCR and fluorescence signal detection were performed using a SmartChip Real-Time PCR System (WaferGen Biosystems, USA), and amplification and dissolution curves were automatically generated. Canco was employed to derive the threshold (Ct) values and detection rates for each gene in a sample, with 16S rRNA serving as the internal reference to normalize the data for the relative quantitative information of each gene. The absolute quantitative information for the 16S rRNA gene was obtained, whereas the absolute quantitative information for the other genes was obtained via conversion. Quality control was performed on the resulting Ct values, and genes satisfying the following conditions were discarded: (i) an amplification efficiency of less than 1.8 or more than 2.2; (ii) an amplified negative control; and (iii) Ct values greater than 31 (considered to indicate no amplification). Finally, the copy number of each gene was calculated as follows: $copy\;number\;=\;10^{(31-Ct)/\left(10/3\right)}.$

### Statistical analysis

The R software package (version 4.2) (55) was used for the data analysis and visualization. Principal component analysis (PCA) and nonmetric multidimensional scaling (NMDS) were employed to assess the differences in the structures of the microbial communities and functional genes on the basis of the Bray–Curtis distance. We also tested the significance of the differences using permutational multivariate analysis of variance (PERMANOVA) (Adonis), analysis of similarities (ANOSIM), and the response permutation procedure (MRPP). Analysis of variance (ANOVA) was used to evaluate the effects of the restoration year on microbial alpha and beta diversity, as well as functional gene diversity. Post hoc comparisons were conducted using Tukey’s honestly significant difference (HSD) test, with a significance level of *P* < 0.05. The PCA, NMDS, Adonis, ANOSIM, MRPP, Mantel tests, alpha diversity, and distance matrices were determined with the “vegan” package. Furthermore, the spatial turnover rate was obtained by fitting the Bray–Curtis distance matrix with soil depth using the “ggplot2” package. ANOVA and Tukey’s HSD tests were performed with the “stats” package, while the letters indicating statistical significance were obtained using the “multcompView” package. The R package “randomForest” was used with default parameters to screen for restoration-discriminant fungal species by random forest classification (56).

To determine the microbial community assembly process, we employed the null model, the β-nearest taxon index (βNTI), and Bray–Curtis-based Raup–Crick (RC_Bray_) data for ecological inference (57). The βNTI and RC_Bray_ values were calculated using the “comdistnt” and “vegan” packages in R, respectively. To understand the assembly process during the restoration process (58), we combined two adjacent restoration years into one restoration stage, resulting in four types of restoration progress: 0 to 5 years, 5 to 15 years, 15 to 28 years, and 28 to 36 years. The rRNA operon (rrn) copy number of each ASV was estimated from the rnnDB database (59). We calculated the abundance-weighted average rRNA operon copy numbers of the ASVs for each sample to assess the microbial life strategies. The co-occurrence network was constructed using the top 500 abundant bacterial and fungal species. We then calculated the SparCC correlation matrix, and edges with a SPARCC correlation coefficient > 0.6 and *P* < 0.01 were selected to build the network. The network construction process utilized the “ggclusternet” package (60).

An SEM was constructed using the “piecewiseSEM” package (61). The soil physical and chemical properties were calculated on the basis of the Euclidean distance matrix, and the microbial structure and function were calculated on the basis of the Bray–Curtis distance matrix. The soil physicochemical properties were represented by a composite variable that included SOC, TN, TP, NO_3_^−^, NH_4_^+^, and pH. Similarly, the assembly mechanism was represented by a composite variable encompassing βNTI, βMNTD, and RCb. The direct path coefficient of a composite variable on the microbial community was taken to represent the direct effect. The indirect path coefficient was determined by identifying all the indirect paths of the composite variable and subsequently calculating the product of the coefficients for each indirect path. The effects of all paths were then aggregated to determine the overall indirect effect of the composite variable.

## Data Availability

The data employed in the study are presented in the paper and/or in the supplemental material. The 16S and ITS sequences were submitted to the NCBI SRA under accession number PRJNA1138184.

## SUPPLEMENTAL MATERIAL

Supplemental Material mbio.02834-24-s0001.docx Figures S1 to S10; Tables S1 to S11.

ASM does not own the copyrights to Supplemental Material that may be linked to, or accessed through, an article. The authors have granted ASM a non-exclusive, world-wide license to publish the Supplemental Material files. Please contact the corresponding author directly for reuse.
