# Supplementary material for: Shifts in fungal communities drive soil profile nutrient cycling during grassland restoration
Source: mBio. 2025 Jan 24;16(3):e02834-24. doi: 10.1128/mbio.02834-24 (PMC11898603; doi:10.1128/mbio.02834-24)
Supplement: Supplemental Material — Figures S1 to S10; Tables S1 to S11. [file mbio.02834-24-s0001.docx]

**Figure S1** Coverage of plant communities during restoration.

**
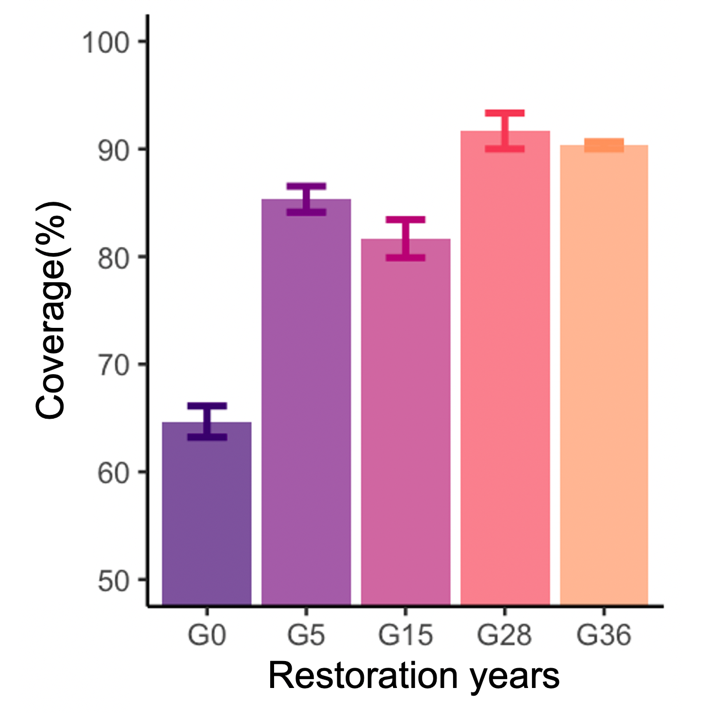
**

**Figure S2** Differences of soil physiochemical properties across soil layers (A) and restoration years (B) based on Bray–Curtis distances. Soil physiochemical properties include soil organic carbon, total nitrogen, total phosphorus, soil NH_4_^+^, NO_3_^-^, and pH.

**
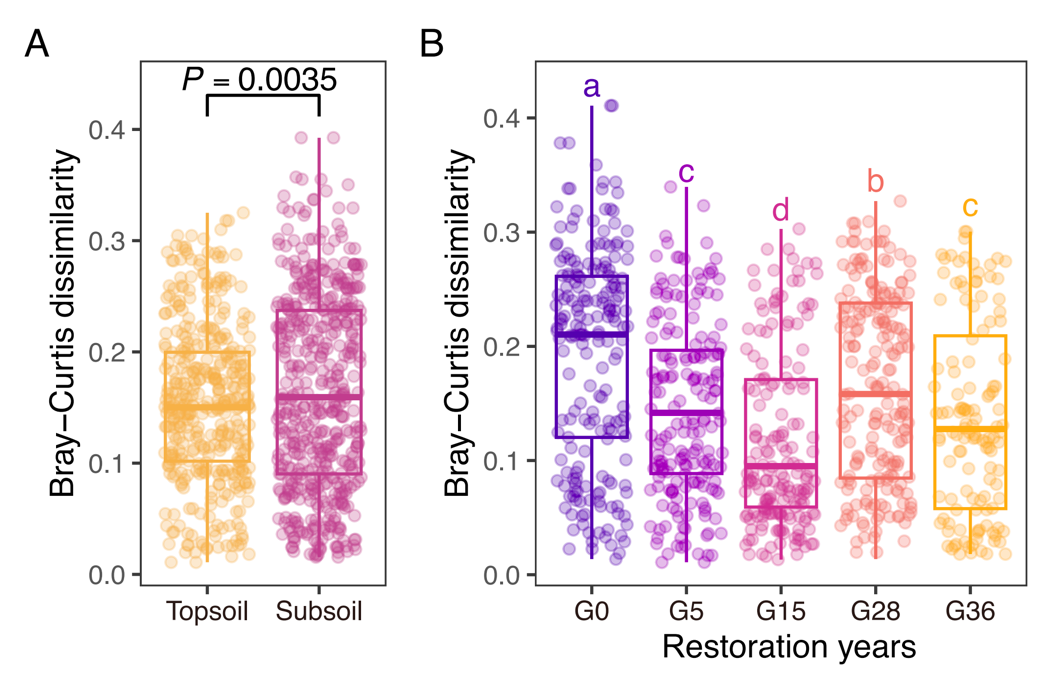
**

**Figure S3** Alpha diversity indices of bacterial and fungal communities across soil depths during restoration.


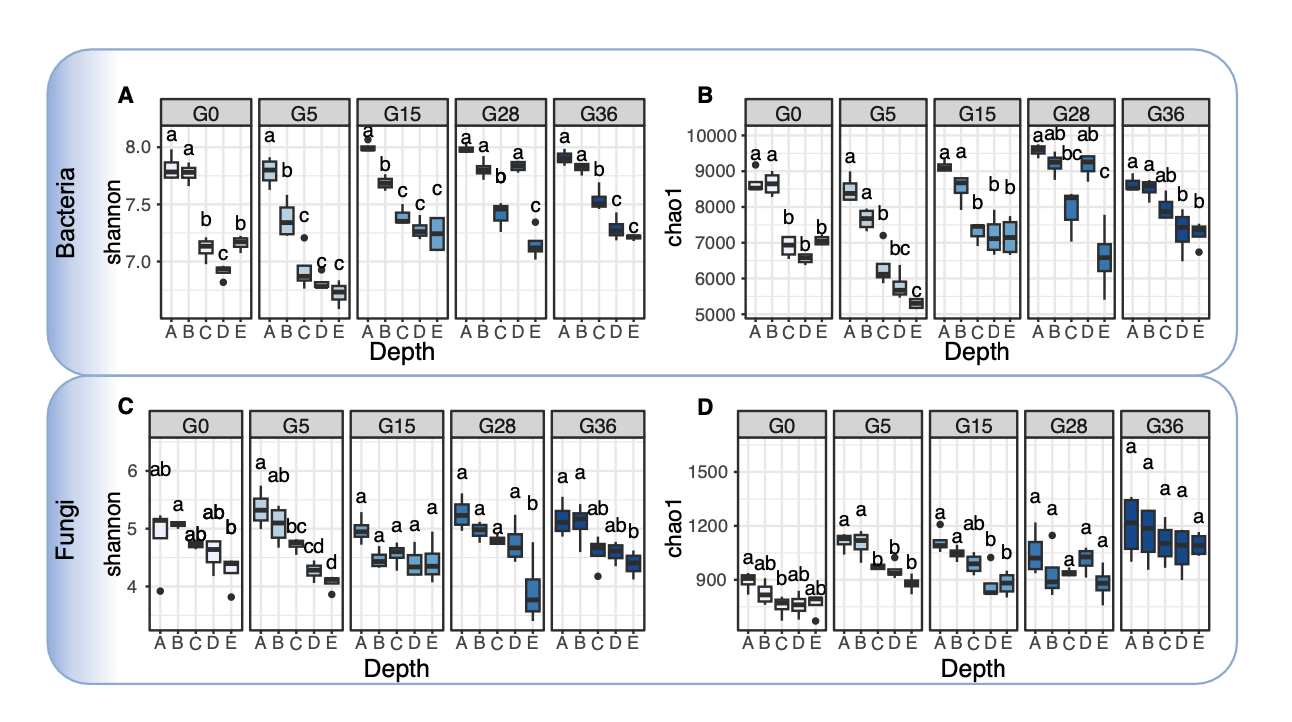


**Figure S4** Significant differences in the R^2^ values between the soil profiles of (A) bacterial and (B) fungal communities during restoration, as shown by Adonis, Anosim, and MRPP tests.


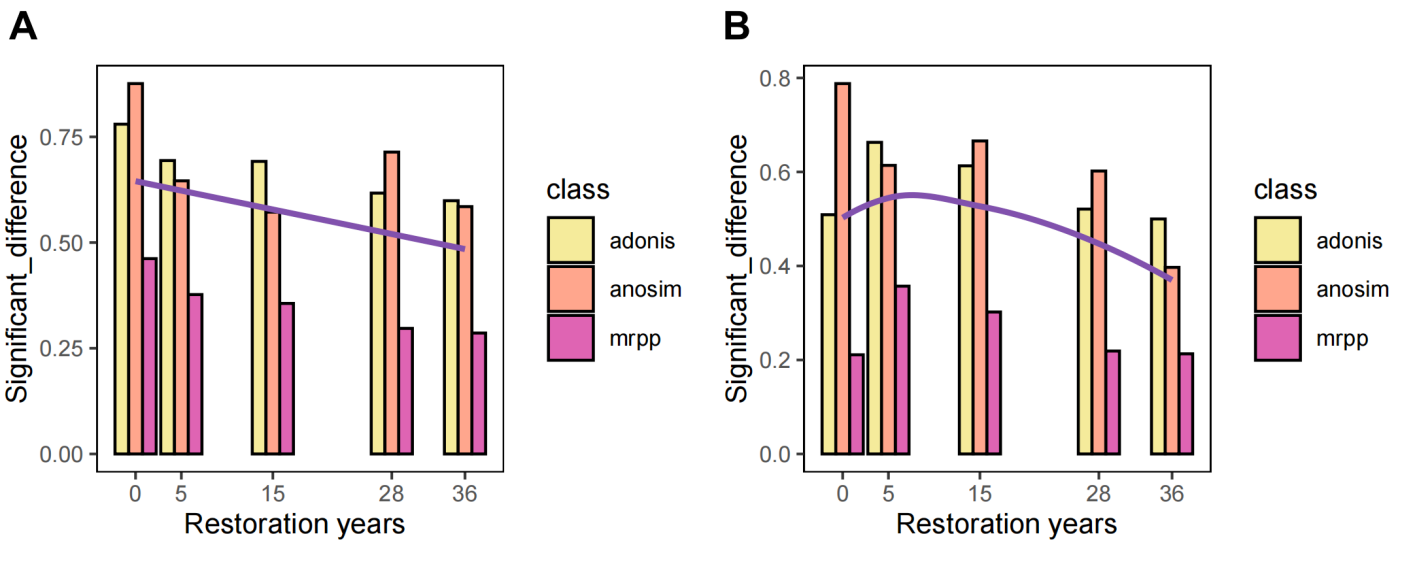


**Figure S5** Venn diagram analysis of the bacterial and fungal community compositions among five restoration stages.

**
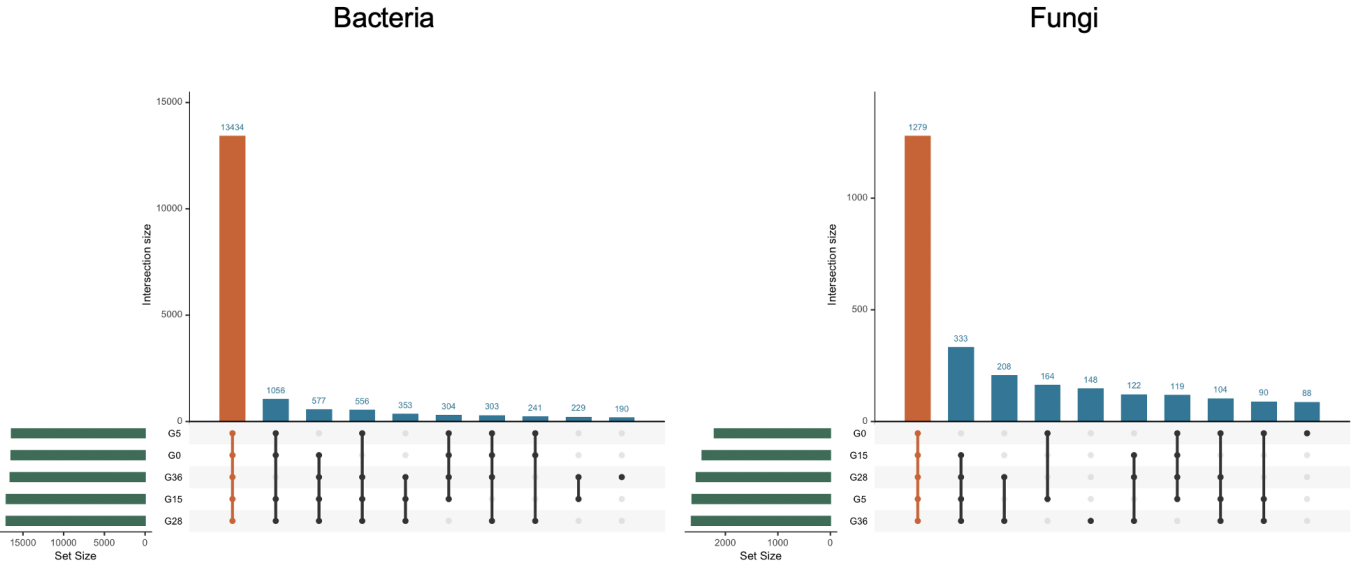
**

**Figure S6** β-NTI and assembly process among the soil layers of the bacterial and fungal communities


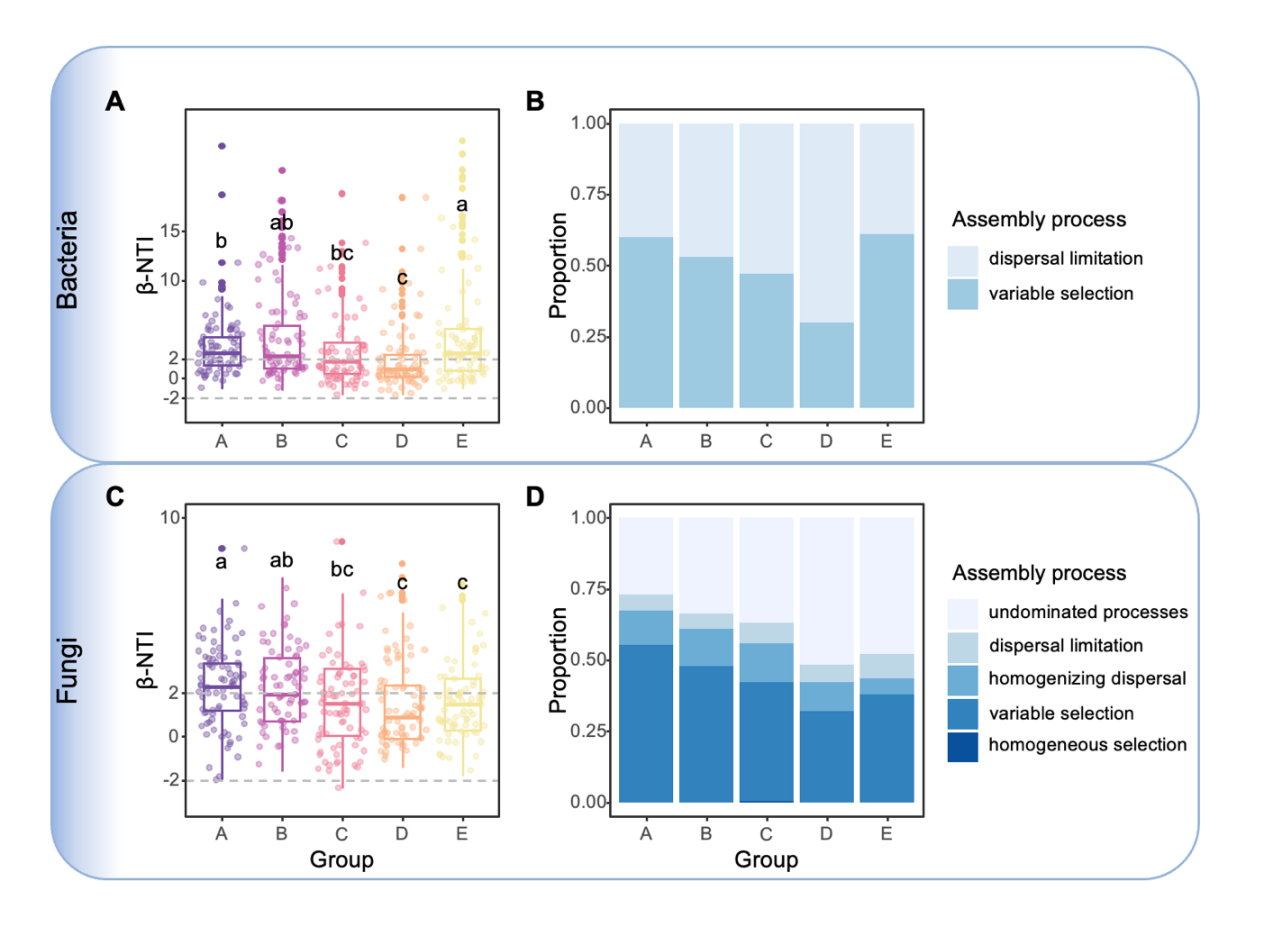


**Figure S7** Bacterial biomass in the sSoil profile bacterial biomass during restoration.

**
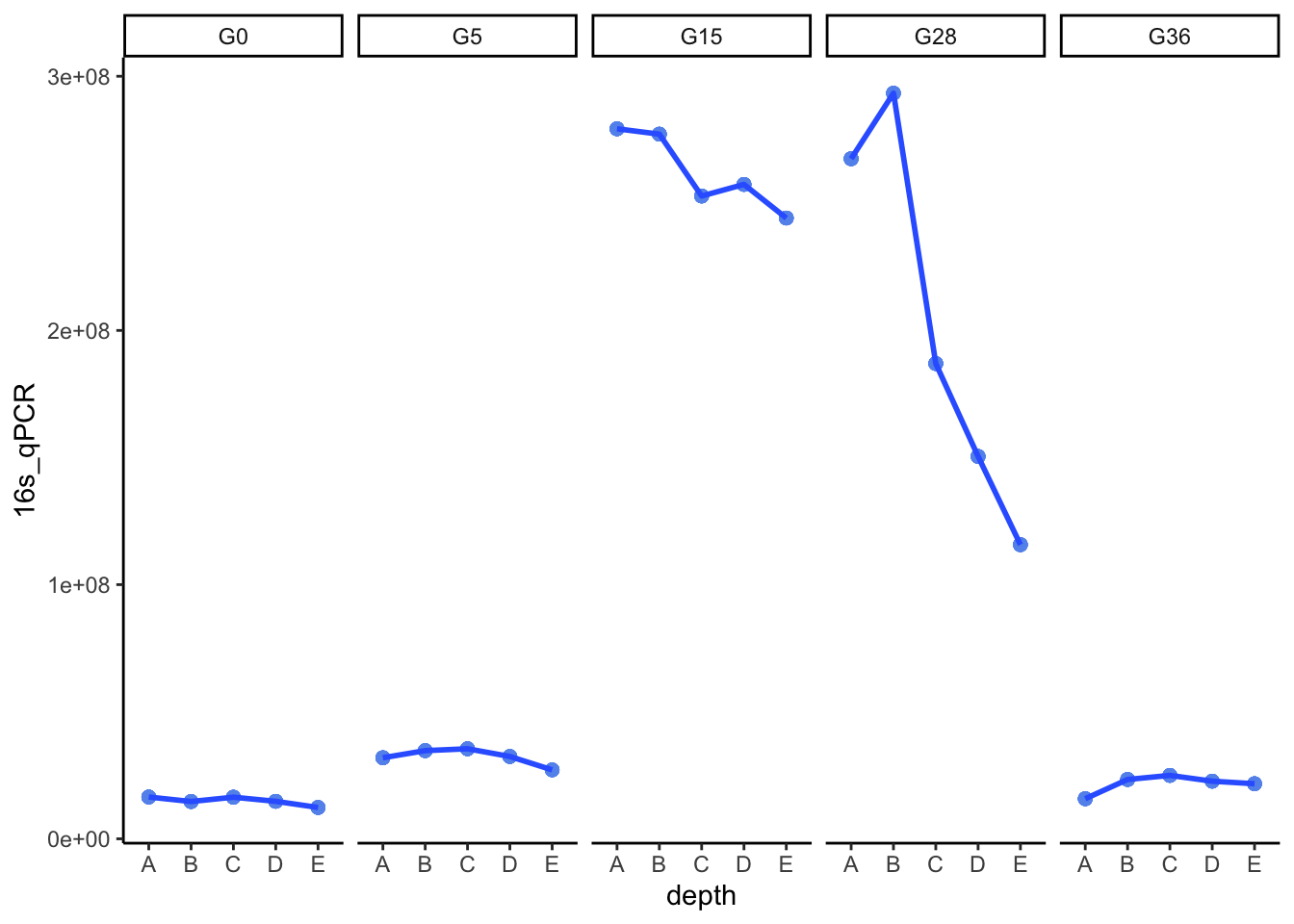
**

**Figure S8** (A) Nonmetric multidimensional scaling (NMDS) analysis and (B) Chao index of the nutrient functional genes based on the Bray–Curtis distance separated by restoration years. (P < 0.001, permutational multivariate analysis of variance (PERMANOVA) by Adonis). Lowercase letters indicate significant differences among restoration years, determined by one-way ANOVA and Duncan's multiple-range test.

**
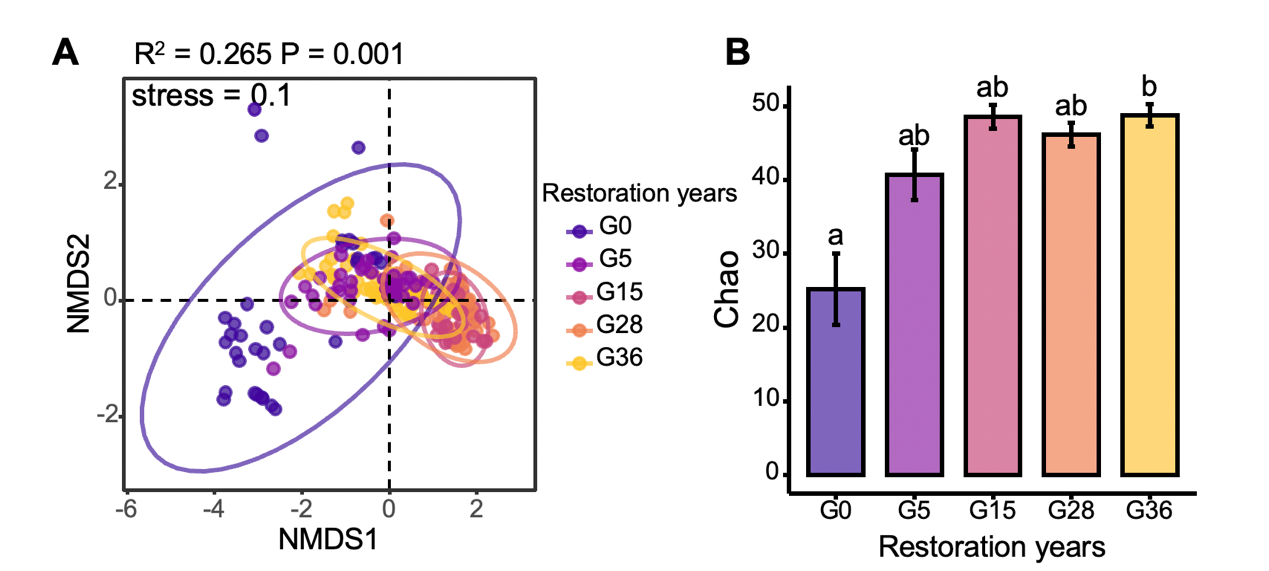
**

**Figure S9** Significant differences in the R^2^ values between the soil profiles of nutrient cycling function genes during restoration, as shown by Adonis, Anosim, and MRPP tests.

**
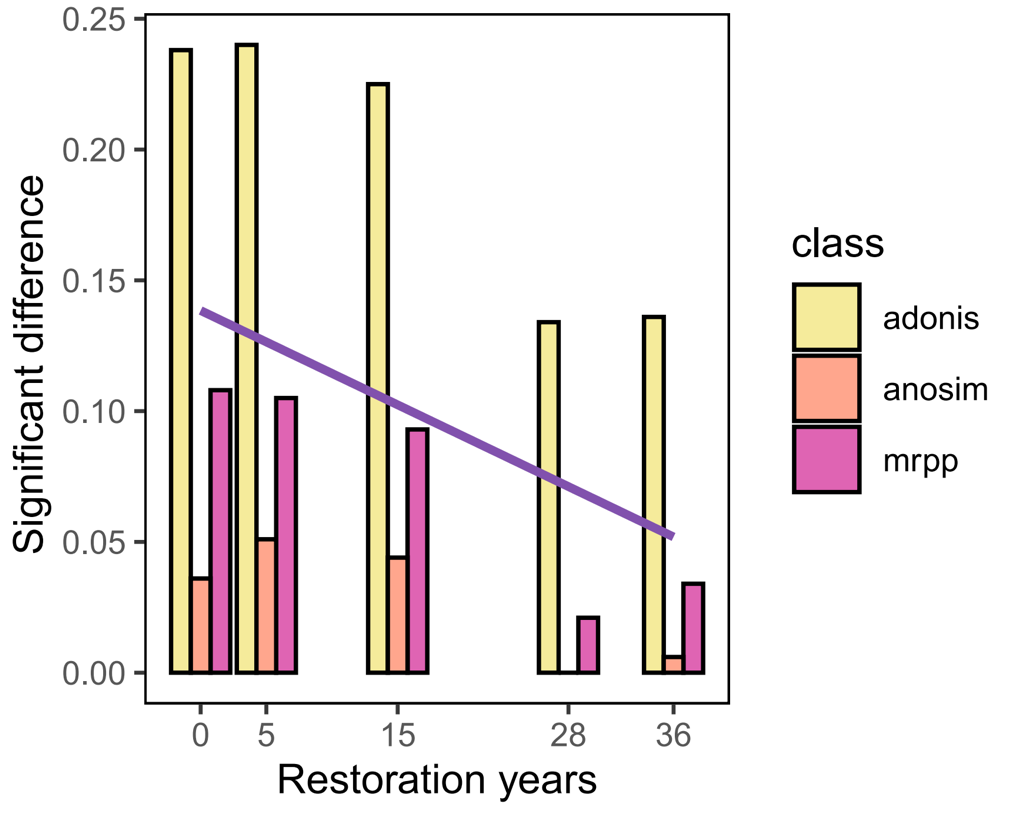
**

**Figure S10** The relative proportions of (A) methane metabolism and (B) the S cycling function between different soil layers during restoration.


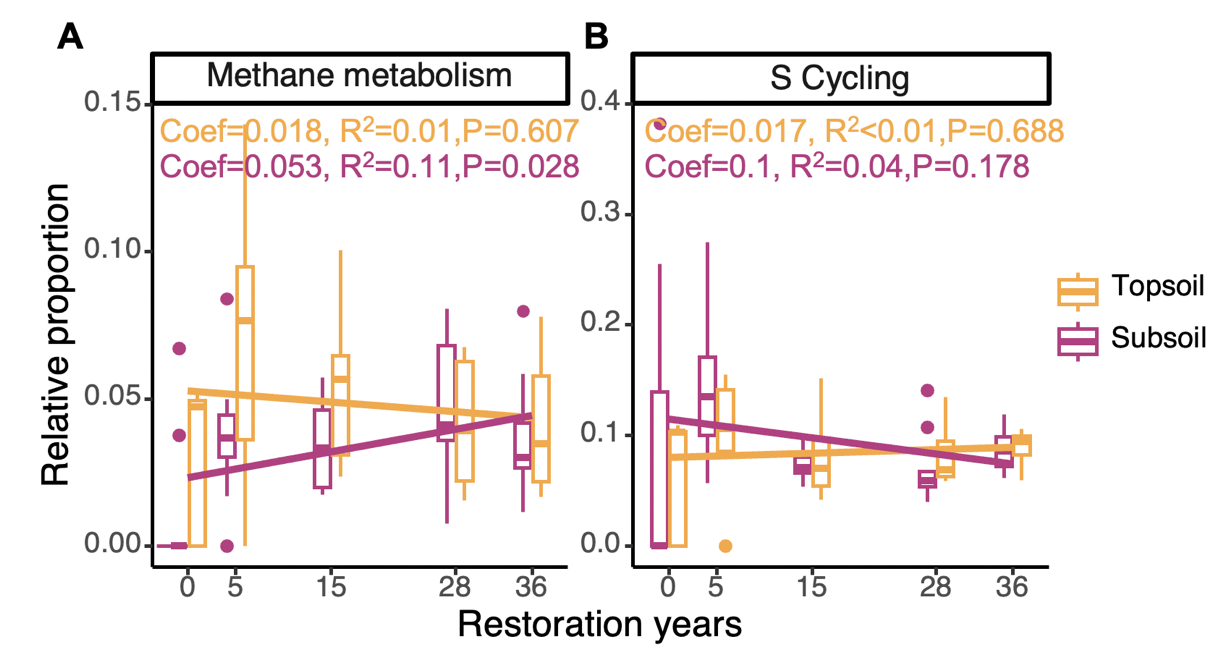


**Table S1** Soil physicochemical properties in the soil profile during restoration.

| Group | SOC (g/Kg) | TN (g/Kg) | TP (g/Kg) | NO_3_^-^ (g/Kg) | NH_4_^+^ (g/Kg) | pH |
| --- | --- | --- | --- | --- | --- | --- |
| G0A | 16.1 (15.9, 16.3) | 1.73 (1.71, 1.75) | 0.70 (0.69, 0.72) | 7.6 (7.3, 10.4) | 3.7 (3.6, 4.1) | 8.30 (8.26, 8.39) |
| G0B | 15.1 (14.9, 15.4) | 1.65 (1.63, 1.65) | 0.69 (0.68, 0.69) | 6.6 (5.9, 7.2) | 4.9 (4.1, 6.4) | 8.30 (8.27, 8.38) |
| G0C | 10.7 (10.6, 10.9) | 1.26 (1.26, 1.27) | 0.57 (0.56, 0.58) | 3.8 (3.4, 4.2) | 11.2 (9.6, 11.8) | 8.34 (8.32, 8.42) |
| G0D | 8.1 (7.9, 8.1) | 0.96 (0.95, 0.96) | 0.55 (0.54, 0.55) | 3.0 (2.8, 3.2) | 6.6 (3.3, 11.1) | 8.49 (8.47, 8.53) |
| G0E | 5.9 (5.7, 6.0) | 0.67 (0.67, 0.68) | 0.50 (0.49, 0.51) | 2.9 (2.4, 3.5) | 3.8 (3.6, 4.3) | 8.50 (8.46, 8.58) |
| G5A | 24.1 (23.6, 24.6) | 2.35 (2.34, 2.37) | 0.66 (0.61, 0.72) | 15.8 (14.3, 17.5) | 13.7 (12.6, 15.6) | 8.30 (8.28, 8.34) |
| G5B | 19.8 (19.4, 20.1) | 2.22 (2.20, 2.23) | 0.69 (0.66, 0.70) | 11.4 (11.0, 11.6) | 8.9 (8.9, 10.9) | 8.36 (8.33, 8.43) |
| G5C | 16.5 (16.1, 16.9) | 1.92 (1.89, 1.95) | 0.66 (0.66, 0.67) | 10.0 (8.5, 14.8) | 8.0 (7.4, 8.4) | 8.37 (8.35, 8.42) |
| G5D | 13.1 (13.0, 13.3) | 1.51 (1.48, 1.54) | 0.64 (0.62, 0.65) | 9.3 (8.8, 10.0) | 8.7 (8.2, 9.2) | 8.42 (8.36, 8.53) |
| G5E | 9.7 (9.4, 9.8) | 1.14 (1.12, 1.16) | 0.68 (0.65, 0.70) | 7.6 (7.3, 7.8) | 10.3 (9.7, 10.8) | 8.48 (8.44, 8.58) |
| G15A | 20.5 (20.4, 20.7) | 2.00 (1.99, 2.03) | 0.74 (0.73, 0.75) | 14.1 (13.1, 14.5) | 4.2 (4.0, 4.5) | 8.09 (8.07, 8.21) |
| G15B | 15.0 (14.7, 15.2) | 1.47 (1.44, 1.49) | 0.68 (0.66, 0.69) | 8.3 (7.8, 8.9) | 7.7 (7.7, 7.7) | 8.28 (8.21, 8.37) |
| G15C | 13.6 (13.4, 13.8) | 1.33 (1.31, 1.35) | 0.67 (0.65, 0.67) | 6.2 (5.7, 8.0) | 9.0 (8.6, 9.9) | 8.42 (8.34, 8.51) |
| G15D | 13.5 (13.3, 13.9) | 1.42 (1.40, 1.46) | 0.73 (0.69, 0.75) | 6.8 (5.9, 8.7) | 9.5 (8.9, 9.8) | 8.35 (8.28, 8.49) |
| G15E | 13.0 (12.9, 13.5) | 1.42 (1.42, 1.43) | 0.76 (0.75, 0.76) | 5.8 (5.0, 6.0) | 11.9 (11.4, 12.2) | 8.55 (8.47, 8.61) |
| G28A | 13.0 (12.5, 13.2) | 1.53 (1.46, 1.60) | 0.68 (0.66, 0.69) | 14.2 (11.6, 15.6) | 8.6 (8.3, 8.8) | 8.51 (8.48, 8.53) |
| G28B | 12.8 (12.8, 13.0) | 1.45 (1.45, 1.46) | 0.64 (0.63, 0.66) | 10.6 (8.9, 12.0) | 12.8 (9.5, 16.6) | 8.54 (8.51, 8.58) |
| G28C | 8.8 (8.5, 9.0) | 1.04 (1.01, 1.06) | 0.59 (0.58, 0.60) | 4.7 (4.3, 5.0) | 15.3 (13.3, 17.6) | 8.59 (8.57, 8.63) |
| G28D | 6.6 (6.6, 6.7) | 0.75 (0.74, 0.77) | 0.52 (0.50, 0.53) | 4.7 (3.3, 6.7) | 14.0 (12.9, 16.3) | 8.66 (8.64, 8.72) |
| G28E | 6.5 (6.2, 6.9) | 0.50 (0.49, 0.51) | 0.44 (0.43, 0.46) | 4.1 (3.7, 4.7) | 12.1 (11.6, 13.2) | 8.66 (8.56, 8.75) |
| G36A | 28.9 (28.2, 29.6) | 3.04 (3.01, 3.08) | 0.69 (0.67, 0.73) | 19.3 (18.2, 20.3) | 8.2 (8.1, 8.3) | 8.35 (8.31, 8.37) |
| G36B | 25.8 (25.5, 26.2) | 2.79 (2.76, 2.81) | 0.71 (0.70, 0.72) | 16.7 (16.0, 17.0) | 8.8 (7.6, 10.4) | 8.30 (8.27, 8.33) |
| G36C | 20.6 (20.4, 20.7) | 2.37 (2.34, 2.39) | 0.71 (0.71, 0.72) | 9.9 (9.5, 10.2) | 8.7 (8.5, 9.1) | 8.45 (8.44, 8.48) |
| G36D | 15.2 (15.0, 15.3) | 1.74 (1.73, 1.76) | 0.58 (0.57, 0.59) | 6.1 (6.0, 6.1) | 7.6 (7.0, 8.3) | 8.56 (8.53, 8.59) |
| G36E | 11.5 (11.2, 11.8) | 1.32 (1.28, 1.36) | 0.54 (0.54, 0.54) | NA (NA, NA) | NA (NA, NA) | 8.59 (8.58, 8.60) |
| p-value | <0.001 | <0.001 | <0.001 | <0.001 | <0.001 | 0.001 |

Note: Kruskal-Wallis rank sum test.

**Table S2** Adonis test of soil physicochemical properties on the basis of the Bray–Curtis distance.

|  | Factor | SumOfSqs | R^2^ | F | Pr(>F) |  |
| --- | --- | --- | --- | --- | --- | --- |
| SOC | restoration | 1.534 | 0.403 | 763.62 | 0.001 | *** |
|  | depth | 1.696 | 0.446 | 844.23 | 0.001 | *** |
|  | restoration:depth | 0.534 | 0.141 | 66.48 | 0.001 | *** |
| TN | restoration | 1.550 | 0.410 | 1657.08 | 0.001 | *** |
|  | depth | 1.556 | 0.412 | 1663.45 | 0.001 | *** |
|  | restoration:depth | 0.656 | 0.174 | 175.27 | 0.001 | *** |
| TP | restoration | 0.146 | 0.304 | 57.69 | 0.001 | *** |
|  | depth | 0.137 | 0.283 | 53.86 | 0.001 | *** |
|  | restoration:depth | 0.151 | 0.314 | 14.94 | 0.001 | *** |
| NH_4_^+^ | restoration | 1.476 | **0.383** | 27.74 | 0.001 | *** |
|  | depth | 0.279 | **0.073** | 5.25 | 0.001 | *** |
|  | restoration:depth | 1.137 | 0.295 | 5.70 | 0.001 | *** |
| NO_3_^-^ | restoration | 1.953 | 0.324 | 33.60 | 0.001 | *** |
|  | depth | 2.293 | 0.381 | 39.44 | 0.001 | *** |
|  | restoration:depth | 0.729 | 0.121 | 3.35 | 0.001 | *** |
| pH | restoration | 0.002 | 0.204 | 7.73 | 0.001 | *** |
|  | depth | 0.003 | 0.256 | 9.69 | 0.001 | *** |
|  | restoration:depth | 0.000 | 0.044 | 0.41 | 0.981 |  |

**Table S3** Adonis test of the bacterial and fungal communities on the basis of the Bray–Curtis distance.

|  |  | | | Bacteria | | | Fungi | | |
| --- | --- | --- | --- | --- | --- | --- | --- | --- | --- |
|  | Df | Sum Of Sqs | R^2^ | F | Pr(>F) | Sum Of Sqs | R^2^ | F | Pr(>F) |
| Restoration year | 4 | 3.66 | 0.25 | 18.52 | 0.001 | 9.22 | 0.30 | 18.36 | 0.001 |
| Depth | 4 | 4.70 | 0.32 | 23.82 | 0.001 | 4.96 | 0.16 | 9.88 | 0.001 |
| Restoration year:Depth | 16 | 2.93 | 0.20 | 3.72 | 0.001 | 6.96 | 0.23 | 3.46 | 0.001 |
| Residual | 73 | 3.60 | 0.24 |  |  | 9.17 | 0.30 |  |  |
| Total | 97 | 14.89 | 1 |  |  | 30.32 | 1 |  |  |

**Table S4** Significant differences among the soil layers of bacterial and fungal communities during restoration, as shown by Adonis, Anosim, and MRPP tests.

|  |  | Bacteria | | Fungi | |
| --- | --- | --- | --- | --- | --- |
| Restoration year | class | Significant difference | P | Significant difference | P |
| G0 | adonis | 0.78 | 0.001 | 0.509 | 0.001 |
| G5 | adonis | 0.694 | 0.001 | 0.663 | 0.001 |
| G15 | adonis | 0.692 | 0.001 | 0.618 | 0.001 |
| G28 | adonis | 0.617 | 0.001 | 0.535 | 0.001 |
| G36 | adonis | 0.599 | 0.001 | 0.5 | 0.001 |
| G0 | mrpp | 0.462 | 0.001 | 0.211 | 0.001 |
| G5 | mrpp | 0.377 | 0.001 | 0.357 | 0.001 |
| G15 | mrpp | 0.356 | 0.001 | 0.301 | 0.001 |
| G28 | mrpp | 0.297 | 0.001 | 0.231 | 0.001 |
| G36 | mrpp | 0.286 | 0.001 | 0.213 | 0.001 |
| G0 | anosim | 0.876 | 0.001 | 0.788 | 0.001 |
| G5 | anosim | 0.646 | 0.001 | 0.614 | 0.001 |
| G15 | anosim | 0.572 | 0.001 | 0.702 | 0.001 |
| G28 | anosim | 0.714 | 0.001 | 0.573 | 0.001 |
| G36 | anosim | 0.585 | 0.001 | 0.397 | 0.001 |

**Table S5** Mantel test between soil physicochemical factors and microbial community during restoration on the basis of the Bray–Curtis distance.

|  | Bacteria | | Fungi | |
| --- | --- | --- | --- | --- |
|  | R^2^ | P | R^2^ | P |
| SOC | 0.5445 | 0.0001 | 0.2576 | 0.0001 |
| TN | 0.5092 | 0.0001 | 0.2163 | 0.0001 |
| TP | 0.2285 | 0.0001 | 0.102 | 0.0034 |
| NH_4_^+^ | **0.0967** | 0.0111 | **0.1864** | 0.0003 |
| NO_3_^-^ | 0.4333 | 0.0001 | 0.2446 | 0.0001 |
| pH | 0.1085 | 0.0012 | 0.0276 | 0.1945 |

**Table S6** Classification of 397 bacterial biomarkers.

| ASV_ID | Mean Decrease Accuracy | Phylum | Class | Order | Family | Genus | Species |
| --- | --- | --- | --- | --- | --- | --- | --- |
| ASV_743 | 9.200 | Proteobacteria | Betaproteobacteria | Unassigned | Unassigned | Unassigned | Unassigned |
| ASV_185 | 8.905 | Actinobacteria | Actinobacteria | Gaiellales | Gaiellaceae | Gaiella | Gaiella_occulta |
| ASV_316 | 8.463 | Actinobacteria | Actinobacteria | Gaiellales | Gaiellaceae | Gaiella | Gaiella_occulta |
| ASV_921 | 7.447 | Actinobacteria | Actinobacteria | Unassigned | Unassigned | Unassigned | Unassigned |
| ASV_464 | 7.407 | Gemmatimonadetes | Gemmatimonadetes | Gemmatimonadales | Gemmatimonadaceae | Gemmatimonas | Gemmatimonas_aurantiaca |
| ASV_2229 | 7.331 | Actinobacteria | Actinobacteria | Actinomycetales | Nocardioidaceae | Kribbella | Unassigned |
| ASV_7161 | 7.210 | Gemmatimonadetes | Gemmatimonadetes | Gemmatimonadales | Gemmatimonadaceae | Gemmatimonas | Gemmatimonas_aurantiaca |
| ASV_855 | 7.180 | Actinobacteria | Actinobacteria | Acidimicrobiales | Unassigned | Unassigned | Unassigned |
| ASV_1299 | 7.140 | Actinobacteria | Unassigned | Unassigned | Unassigned | Unassigned | Unassigned |
| ASV_3664 | 7.134 | Actinobacteria | Actinobacteria | Actinomycetales | Sporichthyaceae | Sporichthya | Sporichthya_brevicatena |
| ASV_643 | 7.084 | Proteobacteria | Gammaproteobacteria | Pseudomonadales | Pseudomonadaceae | Pseudomonas | Unassigned |
| ASV_373 | 6.875 | Actinobacteria | Actinobacteria | Acidimicrobiales | Unassigned | Unassigned | Unassigned |
| ASV_687 | 6.856 | Actinobacteria | Actinobacteria | Unassigned | Unassigned | Unassigned | Unassigned |
| ASV_4062 | 6.835 | Unassigned | Unassigned | Unassigned | Unassigned | Unassigned | Unassigned |
| ASV_693 | 6.833 | Actinobacteria | Actinobacteria | Actinomycetales | Unassigned | Unassigned | Unassigned |
| ASV_5877 | 6.832 | Gemmatimonadetes | Gemmatimonadetes | Gemmatimonadales | Gemmatimonadaceae | Gemmatimonas | Gemmatimonas_aurantiaca |
| ASV_397 | 6.776 | Proteobacteria | Gammaproteobacteria | Pseudomonadales | Pseudomonadaceae | Pseudomonas | Unassigned |
| ASV_3179 | 6.659 | Proteobacteria | Gammaproteobacteria | Unassigned | Unassigned | Unassigned | Unassigned |
| ASV_2233 | 6.645 | Actinobacteria | Actinobacteria | Solirubrobacterales | Solirubrobacteraceae | Solirubrobacter | Unassigned |
| ASV_4617 | 6.640 | Thaumarchaeota | Unassigned | Nitrososphaerales | Nitrososphaeraceae | Nitrososphaera | Unassigned |
| ASV_6294 | 6.592 | Proteobacteria | Alphaproteobacteria | Unassigned | Unassigned | Unassigned | Unassigned |
| ASV_5386 | 6.588 | Acidobacteria | Acidobacteria_Gp6 | Unassigned | Unassigned | Gp6 | Unassigned |
| ASV_3221 | 6.518 | Bacteroidetes | Sphingobacteriia | Sphingobacteriales | Chitinophagaceae | Unassigned | Unassigned |
| ASV_169 | 6.494 | Actinobacteria | Unassigned | Unassigned | Unassigned | Unassigned | Unassigned |
| ASV_1124 | 6.459 | Unassigned | Unassigned | Unassigned | Unassigned | Unassigned | Unassigned |
| ASV_1034 | 6.357 | Unassigned | Unassigned | Unassigned | Unassigned | Unassigned | Unassigned |
| ASV_7164 | 6.352 | Proteobacteria | Alphaproteobacteria | Sphingomonadales | Sphingomonadaceae | Sphingomonas | Sphingomonas_sediminicola |
| ASV_6151 | 6.335 | Unassigned | Unassigned | Unassigned | Unassigned | Unassigned | Unassigned |
| ASV_1775 | 6.264 | Unassigned | Unassigned | Unassigned | Unassigned | Unassigned | Unassigned |
| ASV_3492 | 6.222 | Proteobacteria | Betaproteobacteria | Unassigned | Unassigned | Unassigned | Unassigned |
| ASV_1535 | 6.188 | Actinobacteria | Actinobacteria | Gaiellales | Gaiellaceae | Gaiella | Gaiella_occulta |
| ASV_402 | 6.033 | Actinobacteria | Actinobacteria | Gaiellales | Gaiellaceae | Gaiella | Gaiella_occulta |
| ASV_1506 | 6.029 | Actinobacteria | Actinobacteria | Gaiellales | Gaiellaceae | Gaiella | Gaiella_occulta |
| ASV_1897 | 5.990 | Acidobacteria | Acidobacteria_Gp16 | Unassigned | Unassigned | Gp16 | Unassigned |
| ASV_3609 | 5.982 | Unassigned | Unassigned | Unassigned | Unassigned | Unassigned | Unassigned |
| ASV_1060 | 5.969 | Unassigned | Unassigned | Unassigned | Unassigned | Unassigned | Unassigned |
| ASV_183 | 5.958 | Actinobacteria | Actinobacteria | Gaiellales | Gaiellaceae | Gaiella | Gaiella_occulta |
| ASV_3243 | 5.950 | Acidobacteria | Acidobacteria_Gp7 | Unassigned | Unassigned | Gp7 | Unassigned |
| ASV_627 | 5.947 | Proteobacteria | Betaproteobacteria | Unassigned | Unassigned | Unassigned | Unassigned |
| ASV_6635 | 5.944 | Armatimonadetes | Unassigned | Unassigned | Unassigned | Armatimonadetes_gp4 | Unassigned |
| ASV_568 | 5.843 | Acidobacteria | Acidobacteria_Gp16 | Unassigned | Unassigned | Gp16 | Unassigned |
| ASV_1191 | 5.829 | Unassigned | Unassigned | Unassigned | Unassigned | Unassigned | Unassigned |
| ASV_7057 | 5.819 | Verrucomicrobia | Subdivision3 | Unassigned | Unassigned | Subdivision3_genera_incertae_sedis | Unassigned |
| ASV_775 | 5.786 | Actinobacteria | Actinobacteria | Actinomycetales | Unassigned | Unassigned | Unassigned |
| ASV_2733 | 5.735 | Actinobacteria | Actinobacteria | Actinomycetales | Unassigned | Unassigned | Unassigned |
| ASV_80 | 5.723 | Actinobacteria | Actinobacteria | Actinomycetales | Unassigned | Unassigned | Unassigned |
| ASV_85 | 5.708 | Actinobacteria | Actinobacteria | Gaiellales | Gaiellaceae | Gaiella | Gaiella_occulta |
| ASV_6302 | 5.688 | Bacteroidetes | Cytophagia | Cytophagales | Cytophagaceae | Adhaeribacter | Adhaeribacter_terreus |
| ASV_1449 | 5.680 | Actinobacteria | Actinobacteria | Solirubrobacterales | Unassigned | Unassigned | Unassigned |
| ASV_2919 | 5.665 | Actinobacteria | Actinobacteria | Gaiellales | Gaiellaceae | Gaiella | Gaiella_occulta |
| ASV_3712 | 5.663 | Acidobacteria | Acidobacteria_Gp6 | Unassigned | Unassigned | Gp6 | Unassigned |
| ASV_259 | 5.651 | Gemmatimonadetes | Gemmatimonadetes | Gemmatimonadales | Gemmatimonadaceae | Gemmatimonas | Gemmatimonas_aurantiaca |
| ASV_2353 | 5.641 | Bacteroidetes | Sphingobacteriia | Sphingobacteriales | Unassigned | Unassigned | Unassigned |
| ASV_296 | 5.614 | Actinobacteria | Actinobacteria | Unassigned | Unassigned | Unassigned | Unassigned |
| ASV_4427 | 5.606 | Latescibacteria | Unassigned | Unassigned | Unassigned | Latescibacteria_genera_incertae_sedis | Unassigned |
| ASV_1194 | 5.591 | Actinobacteria | Actinobacteria | Actinomycetales | Unassigned | Unassigned | Unassigned |
| ASV_2818 | 5.543 | Acidobacteria | Unassigned | Unassigned | Unassigned | Unassigned | Unassigned |
| ASV_103 | 5.531 | Actinobacteria | Actinobacteria | Gaiellales | Gaiellaceae | Gaiella | Gaiella_occulta |
| ASV_900 | 5.524 | Bacteroidetes | Sphingobacteriia | Sphingobacteriales | Chitinophagaceae | Unassigned | Unassigned |
| ASV_1710 | 5.520 | Actinobacteria | Actinobacteria | Actinomycetales | Jiangellaceae | Jiangella | Unassigned |
| ASV_2713 | 5.513 | Acidobacteria | Unassigned | Unassigned | Unassigned | Unassigned | Unassigned |
| ASV_2080 | 5.499 | Proteobacteria | Betaproteobacteria | Rhodocyclales | Rhodocyclaceae | Unassigned | Unassigned |
| ASV_6847 | 5.449 | Actinobacteria | Actinobacteria | Actinomycetales | Unassigned | Unassigned | Unassigned |
| ASV_240 | 5.429 | Unassigned | Unassigned | Unassigned | Unassigned | Unassigned | Unassigned |
| ASV_3695 | 5.407 | Actinobacteria | Actinobacteria | Gaiellales | Gaiellaceae | Gaiella | Gaiella_occulta |
| ASV_731 | 5.384 | Acidobacteria | Acidobacteria_Gp10 | Unassigned | Unassigned | Gp10 | Unassigned |
| ASV_425 | 5.364 | Actinobacteria | Actinobacteria | Solirubrobacterales | Solirubrobacteraceae | Solirubrobacter | Unassigned |
| ASV_2626 | 5.363 | Proteobacteria | Betaproteobacteria | Rhodocyclales | Rhodocyclaceae | Unassigned | Unassigned |
| ASV_791 | 5.354 | Bacteroidetes | Sphingobacteriia | Sphingobacteriales | Sphingobacteriaceae | Solitalea | Solitalea_koreensis |
| ASV_562 | 5.322 | Actinobacteria | Actinobacteria | Solirubrobacterales | Solirubrobacteraceae | Solirubrobacter | Unassigned |
| ASV_5755 | 5.314 | Actinobacteria | Actinobacteria | Gaiellales | Gaiellaceae | Gaiella | Gaiella_occulta |
| ASV_1781 | 5.244 | Actinobacteria | Actinobacteria | Solirubrobacterales | Unassigned | Unassigned | Unassigned |
| ASV_1116 | 5.240 | Verrucomicrobia | Spartobacteria | Unassigned | Unassigned | Spartobacteria_genera_incertae_sedis | Unassigned |
| ASV_4837 | 5.223 | Proteobacteria | Betaproteobacteria | Rhodocyclales | Rhodocyclaceae | Methyloversatilis | Methyloversatilis_thermotolerans |
| ASV_4975 | 5.211 | Latescibacteria | Unassigned | Unassigned | Unassigned | Latescibacteria_genera_incertae_sedis | Unassigned |
| ASV_2705 | 5.192 | Bacteroidetes | Sphingobacteriia | Sphingobacteriales | Chitinophagaceae | Unassigned | Unassigned |
| ASV_5322 | 5.175 | Actinobacteria | Actinobacteria | Unassigned | Unassigned | Unassigned | Unassigned |
| ASV_5239 | 5.148 | Actinobacteria | Actinobacteria | Gaiellales | Gaiellaceae | Gaiella | Gaiella_occulta |
| ASV_2365 | 5.116 | Acidobacteria | Acidobacteria_Gp7 | Unassigned | Unassigned | Gp7 | Unassigned |
| ASV_7288 | 5.100 | Actinobacteria | Actinobacteria | Solirubrobacterales | Unassigned | Unassigned | Unassigned |
| ASV_686 | 5.096 | Bacteroidetes | Cytophagia | Cytophagales | Unassigned | Chryseolinea | Chryseolinea_serpens |
| ASV_3982 | 5.070 | Proteobacteria | Gammaproteobacteria | Unassigned | Unassigned | Unassigned | Unassigned |
| ASV_2649 | 5.045 | Proteobacteria | Alphaproteobacteria | Alphaproteobacteria_incertae_sedis | Unassigned | Geminicoccus | Geminicoccus_roseus |
| ASV_5018 | 4.978 | Latescibacteria | Unassigned | Unassigned | Unassigned | Latescibacteria_genera_incertae_sedis | Unassigned |
| ASV_3587 | 4.974 | Actinobacteria | Actinobacteria | Gaiellales | Gaiellaceae | Gaiella | Gaiella_occulta |
| ASV_3404 | 4.971 | Proteobacteria | Unassigned | Unassigned | Unassigned | Unassigned | Unassigned |
| ASV_8075 | 4.940 | Actinobacteria | Actinobacteria | Unassigned | Unassigned | Unassigned | Unassigned |
| ASV_4583 | 4.932 | Proteobacteria | Alphaproteobacteria | Rhodospirillales | Rhodospirillaceae | Unassigned | Unassigned |
| ASV_4009 | 4.924 | Actinobacteria | Actinobacteria | Unassigned | Unassigned | Unassigned | Unassigned |
| ASV_680 | 4.915 | Unassigned | Unassigned | Unassigned | Unassigned | Unassigned | Unassigned |
| ASV_2587 | 4.911 | Bacteroidetes | Sphingobacteriia | Sphingobacteriales | Saprospiraceae | Unassigned | Unassigned |
| ASV_784 | 4.906 | Proteobacteria | Alphaproteobacteria | Sphingomonadales | Sphingomonadaceae | Sphingomonas | Sphingomonas_oryziterrae |
| ASV_1294 | 4.890 | Actinobacteria | Actinobacteria | Acidimicrobiales | Acidimicrobineae_incertae_sedis | Aciditerrimonas | Aciditerrimonas_ferrireducens |
| ASV_1465 | 4.889 | Actinobacteria | Actinobacteria | Gaiellales | Gaiellaceae | Gaiella | Gaiella_occulta |
| ASV_1232 | 4.885 | Proteobacteria | Deltaproteobacteria | Unassigned | Unassigned | Unassigned | Unassigned |
| ASV_3608 | 4.869 | Actinobacteria | Actinobacteria | Unassigned | Unassigned | Unassigned | Unassigned |
| ASV_1134 | 4.865 | Actinobacteria | Actinobacteria | Actinomycetales | Unassigned | Unassigned | Unassigned |
| ASV_6936 | 4.860 | Actinobacteria | Actinobacteria | Acidimicrobiales | Iamiaceae | Aquihabitans | Aquihabitans_daechungensis |
| ASV_1186 | 4.859 | Acidobacteria | Acidobacteria_Gp7 | Unassigned | Unassigned | Gp7 | Unassigned |
| ASV_5483 | 4.826 | Actinobacteria | Actinobacteria | Unassigned | Unassigned | Unassigned | Unassigned |
| ASV_10002 | 4.825 | Proteobacteria | Betaproteobacteria | Burkholderiales | Unassigned | Unassigned | Unassigned |
| ASV_3713 | 4.792 | Acidobacteria | Unassigned | Unassigned | Unassigned | Unassigned | Unassigned |
| ASV_3284 | 4.787 | Proteobacteria | Betaproteobacteria | Burkholderiales | Comamonadaceae | Unassigned | Unassigned |
| ASV_5381 | 4.761 | Proteobacteria | Deltaproteobacteria | Unassigned | Unassigned | Unassigned | Unassigned |
| ASV_3080 | 4.760 | Proteobacteria | Gammaproteobacteria | Unassigned | Unassigned | Unassigned | Unassigned |
| ASV_2197 | 4.721 | Acidobacteria | Acidobacteria_Gp4 | Unassigned | Unassigned | Gp4 | Unassigned |
| ASV_1247 | 4.720 | Acidobacteria | Acidobacteria_Gp16 | Unassigned | Unassigned | Gp16 | Unassigned |
| ASV_646 | 4.711 | Actinobacteria | Actinobacteria | Gaiellales | Gaiellaceae | Gaiella | Gaiella_occulta |
| ASV_1400 | 4.703 | Proteobacteria | Betaproteobacteria | Unassigned | Unassigned | Unassigned | Unassigned |
| ASV_9823 | 4.694 | Actinobacteria | Actinobacteria | Actinomycetales | Streptomycetaceae | Streptomyces | Unassigned |
| ASV_1212 | 4.682 | Unassigned | Unassigned | Unassigned | Unassigned | Unassigned | Unassigned |
| ASV_1348 | 4.674 | Actinobacteria | Actinobacteria | Gaiellales | Gaiellaceae | Gaiella | Gaiella_occulta |
| ASV_5829 | 4.662 | Actinobacteria | Actinobacteria | Gaiellales | Gaiellaceae | Gaiella | Gaiella_occulta |
| ASV_4064 | 4.646 | Proteobacteria | Alphaproteobacteria | Unassigned | Unassigned | Unassigned | Unassigned |
| ASV_1759 | 4.603 | Actinobacteria | Actinobacteria | Acidimicrobiales | Acidimicrobineae_incertae_sedis | Aciditerrimonas | Aciditerrimonas_ferrireducens |
| ASV_2852 | 4.601 | Actinobacteria | Actinobacteria | Actinomycetales | Unassigned | Unassigned | Unassigned |
| ASV_1332 | 4.592 | Actinobacteria | Actinobacteria | Gaiellales | Gaiellaceae | Gaiella | Gaiella_occulta |
| ASV_211 | 4.584 | Actinobacteria | Actinobacteria | Unassigned | Unassigned | Unassigned | Unassigned |
| ASV_4025 | 4.576 | Actinobacteria | Actinobacteria | Solirubrobacterales | Unassigned | Unassigned | Unassigned |
| ASV_2421 | 4.575 | Actinobacteria | Actinobacteria | Solirubrobacterales | Solirubrobacteraceae | Solirubrobacter | Unassigned |
| ASV_638 | 4.562 | Proteobacteria | Alphaproteobacteria | Sphingomonadales | Sphingomonadaceae | Sphingomonas | Sphingomonas_oryziterrae |
| ASV_6559 | 4.558 | Actinobacteria | Actinobacteria | Solirubrobacterales | Solirubrobacteraceae | Solirubrobacter | Unassigned |
| ASV_3655 | 4.552 | Unassigned | Unassigned | Unassigned | Unassigned | Unassigned | Unassigned |
| ASV_4114 | 4.531 | Actinobacteria | Actinobacteria | Actinomycetales | Micromonosporaceae | Unassigned | Unassigned |
| ASV_3464 | 4.530 | Unassigned | Unassigned | Unassigned | Unassigned | Unassigned | Unassigned |
| ASV_4566 | 4.494 | Proteobacteria | Deltaproteobacteria | Unassigned | Unassigned | Unassigned | Unassigned |
| ASV_3813 | 4.472 | Unassigned | Unassigned | Unassigned | Unassigned | Unassigned | Unassigned |
| ASV_779 | 4.442 | Actinobacteria | Actinobacteria | Gaiellales | Gaiellaceae | Gaiella | Gaiella_occulta |
| ASV_5226 | 4.439 | Actinobacteria | Actinobacteria | Solirubrobacterales | Unassigned | Unassigned | Unassigned |
| ASV_5916 | 4.416 | Actinobacteria | Actinobacteria | Solirubrobacterales | Solirubrobacteraceae | Solirubrobacter | Unassigned |
| ASV_109 | 4.409 | Acidobacteria | Acidobacteria_Gp4 | Unassigned | Unassigned | Gp4 | Unassigned |
| ASV_1203 | 4.403 | Actinobacteria | Actinobacteria | Solirubrobacterales | Solirubrobacteraceae | Solirubrobacter | Unassigned |
| ASV_313 | 4.394 | Actinobacteria | Actinobacteria | Gaiellales | Gaiellaceae | Gaiella | Gaiella_occulta |
| ASV_538 | 4.386 | Actinobacteria | Actinobacteria | Gaiellales | Gaiellaceae | Gaiella | Gaiella_occulta |
| ASV_2704 | 4.369 | Unassigned | Unassigned | Unassigned | Unassigned | Unassigned | Unassigned |
| ASV_8369 | 4.367 | Unassigned | Unassigned | Unassigned | Unassigned | Unassigned | Unassigned |
| ASV_1512 | 4.367 | Proteobacteria | Betaproteobacteria | Unassigned | Unassigned | Unassigned | Unassigned |
| ASV_1909 | 4.364 | Acidobacteria | Acidobacteria_Gp16 | Unassigned | Unassigned | Gp16 | Unassigned |
| ASV_577 | 4.362 | Acidobacteria | Acidobacteria_Gp16 | Unassigned | Unassigned | Gp16 | Unassigned |
| ASV_8919 | 4.353 | Bacteroidetes | Unassigned | Unassigned | Unassigned | Unassigned | Unassigned |
| ASV_6147 | 4.349 | Unassigned | Unassigned | Unassigned | Unassigned | Unassigned | Unassigned |
| ASV_2613 | 4.341 | Actinobacteria | Actinobacteria | Gaiellales | Gaiellaceae | Gaiella | Gaiella_occulta |
| ASV_3536 | 4.337 | Acidobacteria | Acidobacteria_Gp16 | Unassigned | Unassigned | Gp16 | Unassigned |
| ASV_4597 | 4.330 | Proteobacteria | Unassigned | Unassigned | Unassigned | Unassigned | Unassigned |
| ASV_58 | 4.327 | Actinobacteria | Actinobacteria | Actinomycetales | Unassigned | Unassigned | Unassigned |
| ASV_4164 | 4.317 | Verrucomicrobia | Spartobacteria | Unassigned | Unassigned | Spartobacteria_genera_incertae_sedis | Unassigned |
| ASV_3396 | 4.317 | Actinobacteria | Actinobacteria | Actinomycetales | Unassigned | Unassigned | Unassigned |
| ASV_9269 | 4.311 | Acidobacteria | Acidobacteria_Gp3 | Unassigned | Unassigned | Gp3 | Unassigned |
| ASV_3777 | 4.302 | Gemmatimonadetes | Gemmatimonadetes | Gemmatimonadales | Gemmatimonadaceae | Gemmatimonas | Gemmatimonas_aurantiaca |
| ASV_1931 | 4.301 | Proteobacteria | Betaproteobacteria | Unassigned | Unassigned | Unassigned | Unassigned |
| ASV_9236 | 4.293 | Unassigned | Unassigned | Unassigned | Unassigned | Unassigned | Unassigned |
| ASV_3195 | 4.292 | Latescibacteria | Unassigned | Unassigned | Unassigned | Latescibacteria_genera_incertae_sedis | Unassigned |
| ASV_6140 | 4.291 | Actinobacteria | Actinobacteria | Actinomycetales | Nocardioidaceae | Aeromicrobium | Unassigned |
| ASV_7172 | 4.286 | Acidobacteria | Unassigned | Unassigned | Unassigned | Unassigned | Unassigned |
| ASV_9082 | 4.279 | Proteobacteria | Deltaproteobacteria | Myxococcales | Unassigned | Unassigned | Unassigned |
| ASV_2064 | 4.278 | Actinobacteria | Actinobacteria | Solirubrobacterales | Unassigned | Unassigned | Unassigned |
| ASV_9584 | 4.271 | Actinobacteria | Actinobacteria | Actinomycetales | Pseudonocardiaceae | Unassigned | Unassigned |
| ASV_7291 | 4.265 | Actinobacteria | Actinobacteria | Solirubrobacterales | Unassigned | Unassigned | Unassigned |
| ASV_3020 | 4.262 | Actinobacteria | Actinobacteria | Gaiellales | Gaiellaceae | Gaiella | Gaiella_occulta |
| ASV_3316 | 4.261 | Actinobacteria | Actinobacteria | Unassigned | Unassigned | Unassigned | Unassigned |
| ASV_10339 | 4.259 | Unassigned | Unassigned | Unassigned | Unassigned | Unassigned | Unassigned |
| ASV_6530 | 4.256 | Actinobacteria | Unassigned | Unassigned | Unassigned | Unassigned | Unassigned |
| ASV_1112 | 4.256 | Actinobacteria | Actinobacteria | Gaiellales | Gaiellaceae | Gaiella | Gaiella_occulta |
| ASV_3189 | 4.211 | Nitrospirae | Nitrospira | Nitrospirales | Nitrospiraceae | Nitrospira | Unassigned |
| ASV_998 | 4.181 | Actinobacteria | Actinobacteria | Gaiellales | Gaiellaceae | Gaiella | Gaiella_occulta |
| ASV_651 | 4.175 | Actinobacteria | Actinobacteria | Gaiellales | Gaiellaceae | Gaiella | Gaiella_occulta |
| ASV_7282 | 4.172 | Actinobacteria | Actinobacteria | Actinomycetales | Unassigned | Unassigned | Unassigned |
| ASV_3346 | 4.162 | Actinobacteria | Actinobacteria | Gaiellales | Gaiellaceae | Gaiella | Gaiella_occulta |
| ASV_2699 | 4.156 | Acidobacteria | Acidobacteria_Gp16 | Unassigned | Unassigned | Gp16 | Unassigned |
| ASV_590 | 4.152 | Actinobacteria | Actinobacteria | Unassigned | Unassigned | Unassigned | Unassigned |
| ASV_2754 | 4.131 | Bacteroidetes | Sphingobacteriia | Sphingobacteriales | Chitinophagaceae | Flavihumibacter | Flavihumibacter_solisilvae |
| ASV_542 | 4.125 | Proteobacteria | Gammaproteobacteria | Pseudomonadales | Pseudomonadaceae | Pseudomonas | Unassigned |
| ASV_500 | 4.116 | Nitrospirae | Nitrospira | Nitrospirales | Nitrospiraceae | Nitrospira | Unassigned |
| ASV_7274 | 4.107 | Proteobacteria | Gammaproteobacteria | Unassigned | Unassigned | Unassigned | Unassigned |
| ASV_942 | 4.101 | Unassigned | Unassigned | Unassigned | Unassigned | Unassigned | Unassigned |
| ASV_2778 | 4.098 | Actinobacteria | Actinobacteria | Acidimicrobiales | Unassigned | Unassigned | Unassigned |
| ASV_6811 | 4.089 | Gemmatimonadetes | Gemmatimonadetes | Gemmatimonadales | Gemmatimonadaceae | Gemmatimonas | Gemmatimonas_aurantiaca |
| ASV_2019 | 4.073 | Actinobacteria | Actinobacteria | Acidimicrobiales | Unassigned | Unassigned | Unassigned |
| ASV_3483 | 4.068 | Unassigned | Unassigned | Unassigned | Unassigned | Unassigned | Unassigned |
| ASV_362 | 4.066 | Actinobacteria | Actinobacteria | Solirubrobacterales | Solirubrobacteraceae | Solirubrobacter | Unassigned |
| ASV_4238 | 4.064 | Unassigned | Unassigned | Unassigned | Unassigned | Unassigned | Unassigned |
| ASV_4455 | 4.051 | Gemmatimonadetes | Gemmatimonadetes | Gemmatimonadales | Gemmatimonadaceae | Gemmatimonas | Gemmatimonas_aurantiaca |
| ASV_603 | 4.050 | Acidobacteria | Acidobacteria_Gp6 | Unassigned | Unassigned | Gp6 | Unassigned |
| ASV_451 | 4.049 | Bacteroidetes | Sphingobacteriia | Sphingobacteriales | Chitinophagaceae | Unassigned | Unassigned |
| ASV_3016 | 4.035 | Acidobacteria | Acidobacteria_Gp6 | Unassigned | Unassigned | Gp6 | Unassigned |
| ASV_10881 | 4.027 | Chloroflexi | Anaerolineae | Anaerolineales | Anaerolineaceae | Unassigned | Unassigned |
| ASV_1880 | 4.027 | Unassigned | Unassigned | Unassigned | Unassigned | Unassigned | Unassigned |
| ASV_3395 | 4.014 | Proteobacteria | Betaproteobacteria | Unassigned | Unassigned | Unassigned | Unassigned |
| ASV_6017 | 4.000 | Proteobacteria | Betaproteobacteria | Rhodocyclales | Rhodocyclaceae | Methyloversatilis | Methyloversatilis_thermotolerans |
| ASV_10217 | 3.994 | Acidobacteria | Acidobacteria_Gp6 | Unassigned | Unassigned | Gp6 | Unassigned |
| ASV_8245 | 3.993 | Bacteroidetes | Flavobacteriia | Flavobacteriales | Flavobacteriaceae | Flavobacterium | Flavobacterium_chungnamense |
| ASV_848 | 3.983 | Bacteroidetes | Cytophagia | Cytophagales | Cytophagaceae | Adhaeribacter | Adhaeribacter_terreus |
| ASV_5279 | 3.974 | Unassigned | Unassigned | Unassigned | Unassigned | Unassigned | Unassigned |
| ASV_7602 | 3.965 | Acidobacteria | Acidobacteria_Gp6 | Unassigned | Unassigned | Gp6 | Unassigned |
| ASV_458 | 3.959 | Unassigned | Unassigned | Unassigned | Unassigned | Unassigned | Unassigned |
| ASV_1075 | 3.953 | Proteobacteria | Gammaproteobacteria | Xanthomonadales | Xanthomonadaceae | Arenimonas | Unassigned |
| ASV_335 | 3.949 | Acidobacteria | Acidobacteria_Gp16 | Unassigned | Unassigned | Gp16 | Unassigned |
| ASV_9888 | 3.928 | Unassigned | Unassigned | Unassigned | Unassigned | Unassigned | Unassigned |
| ASV_2125 | 3.923 | Actinobacteria | Actinobacteria | Actinomycetales | Unassigned | Unassigned | Unassigned |
| ASV_1809 | 3.915 | Acidobacteria | Acidobacteria_Gp16 | Unassigned | Unassigned | Gp16 | Unassigned |
| ASV_5884 | 3.899 | Acidobacteria | Acidobacteria_Gp16 | Unassigned | Unassigned | Gp16 | Unassigned |
| ASV_5032 | 3.896 | Proteobacteria | Deltaproteobacteria | Myxococcales | Unassigned | Unassigned | Unassigned |
| ASV_3355 | 3.893 | Gemmatimonadetes | Gemmatimonadetes | Gemmatimonadales | Gemmatimonadaceae | Gemmatimonas | Gemmatimonas_aurantiaca |
| ASV_3815 | 3.893 | Proteobacteria | Alphaproteobacteria | Rhizobiales | Xanthobacteraceae | Unassigned | Unassigned |
| ASV_750 | 3.878 | Proteobacteria | Betaproteobacteria | Burkholderiales | Unassigned | Unassigned | Unassigned |
| ASV_1057 | 3.875 | Acidobacteria | Acidobacteria_Gp4 | Unassigned | Unassigned | Aridibacter | Unassigned |
| ASV_1081 | 3.867 | Unassigned | Unassigned | Unassigned | Unassigned | Unassigned | Unassigned |
| ASV_2220 | 3.855 | Gemmatimonadetes | Gemmatimonadetes | Gemmatimonadales | Gemmatimonadaceae | Gemmatimonas | Gemmatimonas_aurantiaca |
| ASV_3910 | 3.852 | Proteobacteria | Gammaproteobacteria | Chromatiales | Unassigned | Unassigned | Unassigned |
| ASV_1793 | 3.838 | Bacteroidetes | Cytophagia | Cytophagales | Cytophagaceae | Adhaeribacter | Adhaeribacter_aquaticus |
| ASV_2267 | 3.826 | Actinobacteria | Actinobacteria | Unassigned | Unassigned | Unassigned | Unassigned |
| ASV_1437 | 3.825 | Actinobacteria | Actinobacteria | Gaiellales | Gaiellaceae | Gaiella | Gaiella_occulta |
| ASV_7170 | 3.812 | Actinobacteria | Actinobacteria | Actinomycetales | Mycobacteriaceae | Mycobacterium | Unassigned |
| ASV_1656 | 3.789 | Actinobacteria | Actinobacteria | Gaiellales | Gaiellaceae | Gaiella | Gaiella_occulta |
| ASV_668 | 3.785 | Proteobacteria | Gammaproteobacteria | Xanthomonadales | Xanthomonadaceae | Lysobacter | Lysobacter_brunescens |
| ASV_1664 | 3.781 | Proteobacteria | Alphaproteobacteria | Sphingomonadales | Sphingomonadaceae | Novosphingobium | Unassigned |
| ASV_9123 | 3.757 | Verrucomicrobia | Subdivision3 | Unassigned | Unassigned | Subdivision3_genera_incertae_sedis | Unassigned |
| ASV_2058 | 3.756 | Unassigned | Unassigned | Unassigned | Unassigned | Unassigned | Unassigned |
| ASV_10875 | 3.755 | Bacteroidetes | Sphingobacteriia | Sphingobacteriales | Chitinophagaceae | Terrimonas | Unassigned |
| ASV_725 | 3.754 | Verrucomicrobia | Subdivision3 | Unassigned | Unassigned | Subdivision3_genera_incertae_sedis | Unassigned |
| ASV_1631 | 3.732 | Chloroflexi | Anaerolineae | Anaerolineales | Anaerolineaceae | Unassigned | Unassigned |
| ASV_2021 | 3.728 | Actinobacteria | Actinobacteria | Acidimicrobiales | Acidimicrobineae_incertae_sedis | Aciditerrimonas | Aciditerrimonas_ferrireducens |
| ASV_1141 | 3.715 | Thaumarchaeota | Unassigned | Nitrososphaerales | Nitrososphaeraceae | Nitrososphaera | Unassigned |
| ASV_182 | 3.707 | Actinobacteria | Actinobacteria | Gaiellales | Gaiellaceae | Gaiella | Gaiella_occulta |
| ASV_1284 | 3.706 | Proteobacteria | Betaproteobacteria | Burkholderiales | Alcaligenaceae | Azohydromonas | Unassigned |
| ASV_6759 | 3.702 | Unassigned | Unassigned | Unassigned | Unassigned | Unassigned | Unassigned |
| ASV_5473 | 3.695 | Actinobacteria | Actinobacteria | Gaiellales | Gaiellaceae | Gaiella | Gaiella_occulta |
| ASV_1133 | 3.694 | Actinobacteria | Actinobacteria | Solirubrobacterales | Unassigned | Unassigned | Unassigned |
| ASV_8457 | 3.692 | Acidobacteria | Acidobacteria_Gp6 | Unassigned | Unassigned | Gp6 | Unassigned |
| ASV_3239 | 3.691 | Acidobacteria | Acidobacteria_Gp7 | Unassigned | Unassigned | Gp7 | Unassigned |
| ASV_2211 | 3.686 | Actinobacteria | Actinobacteria | Actinomycetales | Geodermatophilaceae | Modestobacter | Modestobacter_multiseptatus |
| ASV_7292 | 3.685 | Unassigned | Unassigned | Unassigned | Unassigned | Unassigned | Unassigned |
| ASV_4158 | 3.680 | Bacteroidetes | Cytophagia | Cytophagales | Unassigned | Ohtaekwangia | Ohtaekwangia_koreensis |
| ASV_977 | 3.675 | Unassigned | Unassigned | Unassigned | Unassigned | Unassigned | Unassigned |
| ASV_1303 | 3.674 | Actinobacteria | Actinobacteria | Actinomycetales | Unassigned | Unassigned | Unassigned |
| ASV_1481 | 3.655 | Gemmatimonadetes | Gemmatimonadetes | Gemmatimonadales | Gemmatimonadaceae | Gemmatimonas | Gemmatimonas_aurantiaca |
| ASV_1709 | 3.653 | Proteobacteria | Alphaproteobacteria | Rhizobiales | Rhodobiaceae | Unassigned | Unassigned |
| ASV_1844 | 3.651 | Bacteroidetes | Sphingobacteriia | Sphingobacteriales | Chitinophagaceae | Unassigned | Unassigned |
| ASV_733 | 3.651 | Actinobacteria | Actinobacteria | Solirubrobacterales | Solirubrobacteraceae | Solirubrobacter | Unassigned |
| ASV_843 | 3.641 | Proteobacteria | Deltaproteobacteria | Myxococcales | Unassigned | Unassigned | Unassigned |
| ASV_12280 | 3.635 | Proteobacteria | Alphaproteobacteria | Unassigned | Unassigned | Unassigned | Unassigned |
| ASV_1243 | 3.635 | Actinobacteria | Actinobacteria | Unassigned | Unassigned | Unassigned | Unassigned |
| ASV_9149 | 3.632 | Acidobacteria | Acidobacteria_Gp16 | Unassigned | Unassigned | Gp16 | Unassigned |
| ASV_1833 | 3.627 | Actinobacteria | Actinobacteria | Actinomycetales | Glycomycetaceae | Glycomyces | Unassigned |
| ASV_2206 | 3.626 | Proteobacteria | Gammaproteobacteria | Xanthomonadales | Sinobacteraceae | Povalibacter | Povalibacter_uvarum |
| ASV_2465 | 3.625 | Unassigned | Unassigned | Unassigned | Unassigned | Unassigned | Unassigned |
| ASV_3855 | 3.616 | Actinobacteria | Actinobacteria | Unassigned | Unassigned | Unassigned | Unassigned |
| ASV_7165 | 3.602 | Gemmatimonadetes | Gemmatimonadetes | Gemmatimonadales | Gemmatimonadaceae | Gemmatimonas | Gemmatimonas_aurantiaca |
| ASV_933 | 3.602 | Actinobacteria | Actinobacteria | Actinomycetales | Micromonosporaceae | Unassigned | Unassigned |
| ASV_116 | 3.601 | Actinobacteria | Actinobacteria | Gaiellales | Gaiellaceae | Gaiella | Gaiella_occulta |
| ASV_1268 | 3.590 | Acidobacteria | Acidobacteria_Gp4 | Unassigned | Unassigned | Gp4 | Unassigned |
| ASV_7105 | 3.589 | Verrucomicrobia | Subdivision3 | Unassigned | Unassigned | Subdivision3_genera_incertae_sedis | Unassigned |
| ASV_5888 | 3.588 | Acidobacteria | Acidobacteria_Gp6 | Unassigned | Unassigned | Gp6 | Unassigned |
| ASV_413 | 3.586 | Unassigned | Unassigned | Unassigned | Unassigned | Unassigned | Unassigned |
| ASV_981 | 3.582 | Actinobacteria | Actinobacteria | Solirubrobacterales | Unassigned | Unassigned | Unassigned |
| ASV_4793 | 3.578 | Acidobacteria | Acidobacteria_Gp6 | Unassigned | Unassigned | Gp6 | Unassigned |
| ASV_1227 | 3.578 | Actinobacteria | Actinobacteria | Actinomycetales | Unassigned | Unassigned | Unassigned |
| ASV_201 | 3.574 | Actinobacteria | Actinobacteria | Actinomycetales | Unassigned | Unassigned | Unassigned |
| ASV_4588 | 3.548 | Actinobacteria | Actinobacteria | Actinomycetales | Unassigned | Unassigned | Unassigned |
| ASV_1630 | 3.548 | Unassigned | Unassigned | Unassigned | Unassigned | Unassigned | Unassigned |
| ASV_1291 | 3.540 | Actinobacteria | Actinobacteria | Gaiellales | Gaiellaceae | Gaiella | Gaiella_occulta |
| ASV_8718 | 3.532 | Actinobacteria | Actinobacteria | Acidimicrobiales | Acidimicrobineae_incertae_sedis | Aciditerrimonas | Aciditerrimonas_ferrireducens |
| ASV_11172 | 3.527 | Latescibacteria | Unassigned | Unassigned | Unassigned | Latescibacteria_genera_incertae_sedis | Unassigned |
| ASV_7 | 3.526 | Actinobacteria | Actinobacteria | Solirubrobacterales | Solirubrobacteraceae | Solirubrobacter | Unassigned |
| ASV_195 | 3.516 | Unassigned | Unassigned | Unassigned | Unassigned | Unassigned | Unassigned |
| ASV_2906 | 3.515 | Actinobacteria | Actinobacteria | Solirubrobacterales | Solirubrobacteraceae | Solirubrobacter | Unassigned |
| ASV_492 | 3.507 | Actinobacteria | Actinobacteria | Gaiellales | Gaiellaceae | Gaiella | Gaiella_occulta |
| ASV_2752 | 3.500 | Proteobacteria | Alphaproteobacteria | Sphingomonadales | Sphingomonadaceae | Sphingosinicella | Unassigned |
| ASV_228 | 3.497 | Actinobacteria | Actinobacteria | Solirubrobacterales | Solirubrobacteraceae | Solirubrobacter | Unassigned |
| ASV_9629 | 3.495 | Bacteroidetes | Sphingobacteriia | Sphingobacteriales | Chitinophagaceae | Chitinophaga | Chitinophaga_niabensis |
| ASV_1616 | 3.492 | Unassigned | Unassigned | Unassigned | Unassigned | Unassigned | Unassigned |
| ASV_2381 | 3.487 | Actinobacteria | Actinobacteria | Gaiellales | Gaiellaceae | Gaiella | Gaiella_occulta |
| ASV_6848 | 3.485 | Latescibacteria | Unassigned | Unassigned | Unassigned | Latescibacteria_genera_incertae_sedis | Unassigned |
| ASV_2363 | 3.484 | Nitrospirae | Nitrospira | Nitrospirales | Nitrospiraceae | Nitrospira | Unassigned |
| ASV_2599 | 3.482 | Actinobacteria | Unassigned | Unassigned | Unassigned | Unassigned | Unassigned |
| ASV_882 | 3.481 | Actinobacteria | Actinobacteria | Solirubrobacterales | Solirubrobacteraceae | Solirubrobacter | Unassigned |
| ASV_9893 | 3.476 | Proteobacteria | Gammaproteobacteria | Xanthomonadales | Xanthomonadaceae | Dyella | Unassigned |
| ASV_375 | 3.476 | Firmicutes | Negativicutes | Selenomonadales | Acidaminococcaceae | Phascolarctobacterium | Phascolarctobacterium_succinatutens |
| ASV_435 | 3.474 | Actinobacteria | Unassigned | Unassigned | Unassigned | Unassigned | Unassigned |
| ASV_352 | 3.471 | Acidobacteria | Acidobacteria_Gp4 | Unassigned | Unassigned | Gp4 | Unassigned |
| ASV_3290 | 3.460 | Nitrospirae | Nitrospira | Nitrospirales | Nitrospiraceae | Nitrospira | Unassigned |
| ASV_4535 | 3.459 | Actinobacteria | Actinobacteria | Solirubrobacterales | Solirubrobacteraceae | Solirubrobacter | Unassigned |
| ASV_951 | 3.457 | Proteobacteria | Gammaproteobacteria | Chromatiales | Unassigned | Unassigned | Unassigned |
| ASV_507 | 3.450 | Actinobacteria | Actinobacteria | Solirubrobacterales | Solirubrobacteraceae | Solirubrobacter | Unassigned |
| ASV_1651 | 3.446 | Proteobacteria | Betaproteobacteria | Unassigned | Unassigned | Unassigned | Unassigned |
| ASV_3799 | 3.428 | Armatimonadetes | Chthonomonadetes | Chthonomonadales | Chthonomonadaceae | Chthonomonas/Armatimonadetes_gp3 | Unassigned |
| ASV_263 | 3.424 | Actinobacteria | Actinobacteria | Gaiellales | Gaiellaceae | Gaiella | Gaiella_occulta |
| ASV_2178 | 3.413 | Acidobacteria | Acidobacteria_Gp6 | Unassigned | Unassigned | Gp6 | Unassigned |
| ASV_7493 | 3.413 | Unassigned | Unassigned | Unassigned | Unassigned | Unassigned | Unassigned |
| ASV_1359 | 3.409 | Actinobacteria | Actinobacteria | Gaiellales | Gaiellaceae | Gaiella | Gaiella_occulta |
| ASV_6947 | 3.405 | Unassigned | Unassigned | Unassigned | Unassigned | Unassigned | Unassigned |
| ASV_2198 | 3.405 | Proteobacteria | Alphaproteobacteria | Rhodospirillales | Unassigned | Unassigned | Unassigned |
| ASV_4739 | 3.397 | Actinobacteria | Unassigned | Unassigned | Unassigned | Unassigned | Unassigned |
| ASV_3636 | 3.394 | Armatimonadetes | Chthonomonadetes | Chthonomonadales | Chthonomonadaceae | Chthonomonas/Armatimonadetes_gp3 | Unassigned |
| ASV_2462 | 3.393 | Proteobacteria | Betaproteobacteria | Burkholderiales | Burkholderiaceae | Unassigned | Unassigned |
| ASV_50 | 3.383 | Gemmatimonadetes | Gemmatimonadetes | Gemmatimonadales | Gemmatimonadaceae | Gemmatimonas | Gemmatimonas_aurantiaca |
| ASV_4262 | 3.383 | Bacteroidetes | Sphingobacteriia | Sphingobacteriales | Sphingobacteriaceae | Solitalea | Unassigned |
| ASV_7173 | 3.376 | Unassigned | Unassigned | Unassigned | Unassigned | Unassigned | Unassigned |
| ASV_3441 | 3.375 | Actinobacteria | Actinobacteria | Solirubrobacterales | Unassigned | Unassigned | Unassigned |
| ASV_1407 | 3.373 | Proteobacteria | Alphaproteobacteria | Rhizobiales | Unassigned | Unassigned | Unassigned |
| ASV_291 | 3.372 | Actinobacteria | Actinobacteria | Gaiellales | Gaiellaceae | Gaiella | Gaiella_occulta |
| ASV_176 | 3.372 | Proteobacteria | Alphaproteobacteria | Sphingomonadales | Sphingomonadaceae | Sphingomonas | Unassigned |
| ASV_1045 | 3.370 | Acidobacteria | Acidobacteria_Gp16 | Unassigned | Unassigned | Gp16 | Unassigned |
| ASV_5392 | 3.369 | Proteobacteria | Alphaproteobacteria | Rhizobiales | Unassigned | Unassigned | Unassigned |
| ASV_7818 | 3.367 | Unassigned | Unassigned | Unassigned | Unassigned | Unassigned | Unassigned |
| ASV_4387 | 3.366 | Proteobacteria | Gammaproteobacteria | Xanthomonadales | Xanthomonadaceae | Unassigned | Unassigned |
| ASV_2284 | 3.363 | Acidobacteria | Acidobacteria_Gp4 | Unassigned | Unassigned | Gp4 | Unassigned |
| ASV_1963 | 3.362 | Acidobacteria | Acidobacteria_Gp16 | Unassigned | Unassigned | Gp16 | Unassigned |
| ASV_6104 | 3.354 | Unassigned | Unassigned | Unassigned | Unassigned | Unassigned | Unassigned |
| ASV_1357 | 3.353 | Actinobacteria | Actinobacteria | Gaiellales | Gaiellaceae | Gaiella | Gaiella_occulta |
| ASV_1945 | 3.352 | Latescibacteria | Unassigned | Unassigned | Unassigned | Latescibacteria_genera_incertae_sedis | Unassigned |
| ASV_7071 | 3.351 | Proteobacteria | Deltaproteobacteria | Myxococcales | Unassigned | Unassigned | Unassigned |
| ASV_423 | 3.350 | Acidobacteria | Unassigned | Unassigned | Unassigned | Unassigned | Unassigned |
| ASV_6557 | 3.350 | Actinobacteria | Actinobacteria | Gaiellales | Gaiellaceae | Gaiella | Gaiella_occulta |
| ASV_980 | 3.350 | Actinobacteria | Actinobacteria | Gaiellales | Gaiellaceae | Gaiella | Gaiella_occulta |
| ASV_5174 | 3.347 | Unassigned | Unassigned | Unassigned | Unassigned | Unassigned | Unassigned |
| ASV_30 | 3.347 | Thaumarchaeota | Unassigned | Nitrososphaerales | Nitrososphaeraceae | Nitrososphaera | Unassigned |
| ASV_104 | 3.345 | Actinobacteria | Actinobacteria | Gaiellales | Gaiellaceae | Gaiella | Gaiella_occulta |
| ASV_442 | 3.337 | Acidobacteria | Acidobacteria_Gp6 | Unassigned | Unassigned | Gp6 | Unassigned |
| ASV_3967 | 3.337 | Acidobacteria | Acidobacteria_Gp16 | Unassigned | Unassigned | Gp16 | Unassigned |
| ASV_2326 | 3.333 | Actinobacteria | Actinobacteria | Actinomycetales | Micromonosporaceae | Unassigned | Unassigned |
| ASV_3778 | 3.332 | Actinobacteria | Unassigned | Unassigned | Unassigned | Unassigned | Unassigned |
| ASV_1954 | 3.327 | Actinobacteria | Actinobacteria | Unassigned | Unassigned | Unassigned | Unassigned |
| ASV_5761 | 3.324 | Proteobacteria | Alphaproteobacteria | Sphingomonadales | Sphingomonadaceae | Sphingomonas | Unassigned |
| ASV_4438 | 3.324 | Actinobacteria | Actinobacteria | Solirubrobacterales | Unassigned | Unassigned | Unassigned |
| ASV_4652 | 3.322 | Actinobacteria | Actinobacteria | Solirubrobacterales | Unassigned | Unassigned | Unassigned |
| ASV_8244 | 3.315 | Proteobacteria | Betaproteobacteria | Unassigned | Unassigned | Unassigned | Unassigned |
| ASV_11460 | 3.311 | Proteobacteria | Alphaproteobacteria | Sphingomonadales | Sphingomonadaceae | Unassigned | Unassigned |
| ASV_7284 | 3.307 | Acidobacteria | Acidobacteria_Gp16 | Unassigned | Unassigned | Gp16 | Unassigned |
| ASV_3539 | 3.305 | Proteobacteria | Gammaproteobacteria | Unassigned | Unassigned | Unassigned | Unassigned |
| ASV_7601 | 3.303 | Acidobacteria | Acidobacteria_Gp6 | Unassigned | Unassigned | Gp6 | Unassigned |
| ASV_2726 | 3.300 | Acidobacteria | Acidobacteria_Gp16 | Unassigned | Unassigned | Gp16 | Unassigned |
| ASV_2450 | 3.300 | Actinobacteria | Actinobacteria | Gaiellales | Gaiellaceae | Gaiella | Gaiella_occulta |
| ASV_9871 | 3.297 | Unassigned | Unassigned | Unassigned | Unassigned | Unassigned | Unassigned |
| ASV_7720 | 3.285 | Unassigned | Unassigned | Unassigned | Unassigned | Unassigned | Unassigned |
| ASV_9261 | 3.284 | Actinobacteria | Actinobacteria | Unassigned | Unassigned | Unassigned | Unassigned |
| ASV_516 | 3.279 | Unassigned | Unassigned | Unassigned | Unassigned | Unassigned | Unassigned |
| ASV_6301 | 3.277 | Actinobacteria | Actinobacteria | Solirubrobacterales | Unassigned | Unassigned | Unassigned |
| ASV_1172 | 3.277 | Proteobacteria | Betaproteobacteria | Burkholderiales | Unassigned | Unassigned | Unassigned |
| ASV_4699 | 3.268 | Unassigned | Unassigned | Unassigned | Unassigned | Unassigned | Unassigned |
| ASV_1252 | 3.265 | Actinobacteria | Actinobacteria | Unassigned | Unassigned | Unassigned | Unassigned |
| ASV_3188 | 3.256 | Actinobacteria | Actinobacteria | Gaiellales | Gaiellaceae | Gaiella | Gaiella_occulta |
| ASV_47 | 3.249 | Actinobacteria | Actinobacteria | Solirubrobacterales | Unassigned | Unassigned | Unassigned |
| ASV_9188 | 3.240 | Proteobacteria | Alphaproteobacteria | Rhizobiales | Phyllobacteriaceae | Mesorhizobium | Unassigned |
| ASV_3683 | 3.239 | Unassigned | Unassigned | Unassigned | Unassigned | Unassigned | Unassigned |
| ASV_14 | 3.238 | Gemmatimonadetes | Gemmatimonadetes | Gemmatimonadales | Gemmatimonadaceae | Gemmatimonas | Gemmatimonas_aurantiaca |
| ASV_18156 | 3.238 | Acidobacteria | Acidobacteria_Gp6 | Unassigned | Unassigned | Gp6 | Unassigned |
| ASV_1404 | 3.235 | Acidobacteria | Acidobacteria_Gp4 | Unassigned | Unassigned | Gp4 | Unassigned |
| ASV_3907 | 3.230 | Proteobacteria | Deltaproteobacteria | Myxococcales | Unassigned | Unassigned | Unassigned |
| ASV_245 | 3.227 | Acidobacteria | Acidobacteria_Gp4 | Unassigned | Unassigned | Gp4 | Unassigned |
| ASV_9077 | 3.226 | Unassigned | Unassigned | Unassigned | Unassigned | Unassigned | Unassigned |
| ASV_7273 | 3.222 | Bacteroidetes | Sphingobacteriia | Sphingobacteriales | Sphingobacteriaceae | Unassigned | Unassigned |
| ASV_6756 | 3.218 | Verrucomicrobia | Subdivision3 | Unassigned | Unassigned | Subdivision3_genera_incertae_sedis | Unassigned |
| ASV_2913 | 3.217 | Actinobacteria | Unassigned | Unassigned | Unassigned | Unassigned | Unassigned |
| ASV_4068 | 3.216 | Verrucomicrobia | Subdivision3 | Unassigned | Unassigned | Subdivision3_genera_incertae_sedis | Unassigned |
| ASV_4614 | 3.208 | Proteobacteria | Betaproteobacteria | Burkholderiales | Oxalobacteraceae | Unassigned | Unassigned |
| ASV_299 | 3.205 | Firmicutes | Erysipelotrichia | Erysipelotrichales | Erysipelotrichaceae | Allobaculum | Allobaculum_stercoricanis |
| ASV_2376 | 3.205 | Latescibacteria | Unassigned | Unassigned | Unassigned | Latescibacteria_genera_incertae_sedis | Unassigned |
| ASV_9669 | 3.203 | Chloroflexi | Anaerolineae | Anaerolineales | Anaerolineaceae | Unassigned | Unassigned |
| ASV_827 | 3.201 | Actinobacteria | Actinobacteria | Solirubrobacterales | Solirubrobacteraceae | Solirubrobacter | Unassigned |
| ASV_1613 | 3.199 | Actinobacteria | Actinobacteria | Solirubrobacterales | Unassigned | Unassigned | Unassigned |
| ASV_2711 | 3.194 | Actinobacteria | Actinobacteria | Actinomycetales | Intrasporangiaceae | Unassigned | Unassigned |
| ASV_5191 | 3.193 | Actinobacteria | Actinobacteria | Unassigned | Unassigned | Unassigned | Unassigned |
| ASV_1238 | 3.190 | Actinobacteria | Actinobacteria | Actinomycetales | Unassigned | Unassigned | Unassigned |
| ASV_1015 | 3.184 | Unassigned | Unassigned | Unassigned | Unassigned | Unassigned | Unassigned |
| ASV_3198 | 3.183 | Actinobacteria | Actinobacteria | Unassigned | Unassigned | Unassigned | Unassigned |
| ASV_2444 | 3.181 | Actinobacteria | Actinobacteria | Gaiellales | Gaiellaceae | Gaiella | Gaiella_occulta |
| ASV_72 | 3.177 | Actinobacteria | Actinobacteria | Gaiellales | Gaiellaceae | Gaiella | Gaiella_occulta |
| ASV_293 | 3.175 | Actinobacteria | Actinobacteria | Gaiellales | Gaiellaceae | Gaiella | Gaiella_occulta |
| ASV_206 | 3.167 | Actinobacteria | Actinobacteria | Actinomycetales | Nocardioidaceae | Kribbella | Unassigned |
| ASV_5759 | 3.163 | Proteobacteria | Betaproteobacteria | Rhodocyclales | Rhodocyclaceae | Unassigned | Unassigned |
| ASV_7810 | 3.159 | Nitrospirae | Nitrospira | Nitrospirales | Nitrospiraceae | Nitrospira | Unassigned |
| ASV_6569 | 3.154 | Actinobacteria | Actinobacteria | Actinomycetales | Unassigned | Unassigned | Unassigned |
| ASV_6542 | 3.149 | Acidobacteria | Acidobacteria_Gp25 | Unassigned | Unassigned | Gp25 | Unassigned |
| ASV_7719 | 3.140 | Gemmatimonadetes | Gemmatimonadetes | Gemmatimonadales | Gemmatimonadaceae | Gemmatimonas | Gemmatimonas_aurantiaca |
| ASV_14947 | 3.137 | Firmicutes | Bacilli | Bacillales | Bacillaceae_1 | Bacillus | Unassigned |
| ASV_8226 | 3.135 | Proteobacteria | Alphaproteobacteria | Rhizobiales | Hyphomicrobiaceae | Unassigned | Unassigned |
| ASV_1120 | 3.133 | Acidobacteria | Acidobacteria_Gp1 | Unassigned | Unassigned | Gp1 | Unassigned |
| ASV_2330 | 3.132 | Unassigned | Unassigned | Unassigned | Unassigned | Unassigned | Unassigned |
| ASV_5396 | 3.121 | Acidobacteria | Acidobacteria_Gp6 | Unassigned | Unassigned | Gp6 | Unassigned |
| ASV_11764 | 3.120 | Latescibacteria | Unassigned | Unassigned | Unassigned | Latescibacteria_genera_incertae_sedis | Unassigned |
| ASV_4056 | 3.119 | Actinobacteria | Actinobacteria | Gaiellales | Gaiellaceae | Gaiella | Gaiella_occulta |
| ASV_9728 | 3.119 | Acidobacteria | Unassigned | Unassigned | Unassigned | Unassigned | Unassigned |
| ASV_2784 | 3.112 | Actinobacteria | Actinobacteria | Actinomycetales | Streptomycetaceae | Streptomyces | Unassigned |
| ASV_2129 | 3.104 | Acidobacteria | Acidobacteria_Gp16 | Unassigned | Unassigned | Gp16 | Unassigned |
| ASV_5228 | 3.100 | Latescibacteria | Unassigned | Unassigned | Unassigned | Latescibacteria_genera_incertae_sedis | Unassigned |
| ASV_6830 | 3.098 | Unassigned | Unassigned | Unassigned | Unassigned | Unassigned | Unassigned |
| ASV_6142 | 3.092 | Actinobacteria | Actinobacteria | Gaiellales | Gaiellaceae | Gaiella | Gaiella_occulta |
| ASV_13498 | 3.087 | Chloroflexi | Anaerolineae | Anaerolineales | Anaerolineaceae | Unassigned | Unassigned |
| ASV_1629 | 3.087 | Actinobacteria | Actinobacteria | Gaiellales | Gaiellaceae | Gaiella | Gaiella_occulta |
| ASV_6760 | 3.087 | Gemmatimonadetes | Gemmatimonadetes | Gemmatimonadales | Gemmatimonadaceae | Gemmatimonas | Gemmatimonas_aurantiaca |
| ASV_3376 | 3.079 | Proteobacteria | Betaproteobacteria | Unassigned | Unassigned | Unassigned | Unassigned |
| ASV_2493 | 3.071 | Proteobacteria | Deltaproteobacteria | Unassigned | Unassigned | Unassigned | Unassigned |
| ASV_3409 | 3.067 | Latescibacteria | Unassigned | Unassigned | Unassigned | Latescibacteria_genera_incertae_sedis | Unassigned |
| ASV_4822 | 3.065 | Acidobacteria | Acidobacteria_Gp16 | Unassigned | Unassigned | Gp16 | Unassigned |
| ASV_4139 | 3.064 | Acidobacteria | Acidobacteria_Gp6 | Unassigned | Unassigned | Gp6 | Unassigned |
| ASV_756 | 3.051 | Unassigned | Unassigned | Unassigned | Unassigned | Unassigned | Unassigned |

**Table S7** Classification of 131 fungal biomarkers. The fungi guild information is predicted by FUNguild database.

|  | Mean Decrease Accuracy | Phylum | Class | guild |
| --- | --- | --- | --- | --- |
| ASV_298 | 12.48532732 | Ascomycota | Dothideomycetes | NA |
| ASV_110 | 11.91584168 | Ascomycota | Dothideomycetes | NA |
| ASV_68 | 11.51336606 | Ascomycota | Eurotiomycetes | NA |
| ASV_1787 | 11.33740707 | Ascomycota | Geoglossomycetes | Undefined Saprotroph |
| ASV_39 | 11.24845672 | Ascomycota | Unassigned | NA |
| ASV_120 | 10.93245504 | Ascomycota | Eurotiomycetes | NA |
| ASV_151 | 10.85841601 | Ascomycota | Eurotiomycetes | NA |
| ASV_786 | 10.57368685 | Ascomycota | Eurotiomycetes | NA |
| ASV_146 | 10.51666902 | Ascomycota | Leotiomycetes | Plant Pathogen |
| **ASV_705** | **10.18712425** | **Ascomycota** | **Pezizomycetes** | **Dung Saprotroph-Ectomycorrhizal-Soil Saprotroph-Wood Saprotroph** |
| ASV_42 | 10.04518756 | Ascomycota | Eurotiomycetes | NA |
| ASV_202 | 9.98359968 | Unassigned | Unassigned | NA |
| ASV_41 | 9.822573086 | Ascomycota | Eurotiomycetes | Animal Pathogen-Fungal Parasite-Undefined Saprotroph |
| ASV_390 | 9.769432644 | Ascomycota | Leotiomycetes | NA |
| ASV_55 | 9.736549003 | Ascomycota | Eurotiomycetes | NA |
| ASV_161 | 9.723845064 | Ascomycota | Eurotiomycetes | NA |
| ASV_275 | 9.641664846 | Ascomycota | Dothideomycetes | Fungal Parasite-Plant Pathogen-Plant Saprotroph |
| ASV_4 | 9.628823425 | Ascomycota | Eurotiomycetes | NA |
| ASV_524 | 9.60932913 | Ascomycota | Eurotiomycetes | NA |
| ASV_248 | 9.590575487 | Ascomycota | Eurotiomycetes | NA |
| ASV_156 | 9.558006625 | Ascomycota | Eurotiomycetes | NA |
| ASV_612 | 9.531095389 | Unassigned | Unassigned | NA |
| ASV_349 | 9.423154791 | Ascomycota | Dothideomycetes | Undefined Saprotroph |
| ASV_239 | 9.32976012 | Ascomycota | Sordariomycetes | NA |
| ASV_23 | 9.291562431 | Ascomycota | Eurotiomycetes | NA |
| ASV_13 | 9.27566929 | Ascomycota | Unassigned | NA |
| ASV_111 | 9.250733167 | Ascomycota | Sordariomycetes | NA |
| ASV_126 | 9.11111438 | Ascomycota | Unassigned | NA |
| ASV_1262 | 9.057915108 | Ascomycota | Eurotiomycetes | NA |
| ASV_576 | 9.037760119 | Ascomycota | Eurotiomycetes | NA |
| ASV_193 | 8.910084572 | Ascomycota | Sordariomycetes | NA |
| ASV_58 | 8.843861628 | Ascomycota | Unassigned | NA |
| ASV_167 | 8.837675891 | Ascomycota | Leotiomycetes | Undefined Saprotroph |
| ASV_87 | 8.655212625 | Ascomycota | Eurotiomycetes | NA |
| ASV_1344 | 8.645486191 | Ascomycota | Sordariomycetes | Undefined Saprotroph |
| ASV_235 | 8.603364537 | Unassigned | Unassigned | NA |
| ASV_448 | 8.586586163 | Ascomycota | Dothideomycetes | Fungal Parasite-Plant Pathogen-Plant Saprotroph |
| ASV_132 | 8.561068227 | Ascomycota | Eurotiomycetes | NA |
| ASV_103 | 8.552221118 | Ascomycota | Eurotiomycetes | NA |
| ASV_733 | 8.480717499 | Ascomycota | Leotiomycetes | Undefined Saprotroph |
| ASV_57 | 8.425028261 | Ascomycota | Eurotiomycetes | NA |
| ASV_894 | 8.373311789 | Ascomycota | Eurotiomycetes | NA |
| ASV_15 | 8.359948235 | Ascomycota | Eurotiomycetes | NA |
| ASV_436 | 8.34734868 | Ascomycota | Leotiomycetes | Plant Saprotroph-Wood Saprotroph |
| ASV_236 | 8.303411147 | Ascomycota | Sordariomycetes | NA |
| ASV_71 | 8.291836343 | Unassigned | Unassigned | NA |
| **ASV_3098** | **8.278160949** | **Glomeromycota** | **Glomeromycetes** | **Arbuscular Mycorrhizal** |
| ASV_197 | 8.272268309 | Ascomycota | Eurotiomycetes | Dung Saprotroph-Soil Saprotroph |
| ASV_706 | 8.198881817 | Ascomycota | Dothideomycetes | NA |
| ASV_44 | 8.18060852 | Ascomycota | Geoglossomycetes | Undefined Saprotroph |
| ASV_2032 | 8.170144835 | Unassigned | Unassigned | NA |
| ASV_66 | 8.078555069 | Ascomycota | Leotiomycetes | NA |
| ASV_48 | 8.037966809 | Unassigned | Unassigned | NA |
| ASV_10 | 7.965570739 | Ascomycota | Eurotiomycetes | NA |
| ASV_226 | 7.898066165 | Ascomycota | Leotiomycetes | Undefined Saprotroph |
| ASV_76 | 7.891919185 | Ascomycota | Leotiomycetes | Undefined Saprotroph |
| ASV_541 | 7.850946907 | Ascomycota | Eurotiomycetes | NA |
| ASV_131 | 7.799768111 | Unassigned | Unassigned | NA |
| ASV_178 | 7.778349278 | Ascomycota | Eurotiomycetes | NA |
| ASV_592 | 7.764744662 | Basidiomycota | Tremellomycetes | NA |
| ASV_9 | 7.739142106 | Ascomycota | Orbiliomycetes | Wood Saprotroph |
| ASV_28 | 7.716432556 | Ascomycota | Leotiomycetes | NA |
| ASV_153 | 7.601948531 | Ascomycota | Dothideomycetes | NA |
| ASV_19 | 7.498798916 | Ascomycota | Eurotiomycetes | NA |
| ASV_696 | 7.490737606 | Ascomycota | Dothideomycetes | NA |
| ASV_98 | 7.480534357 | Ascomycota | Eurotiomycetes | NA |
| ASV_1284 | 7.464387856 | Ascomycota | Unassigned | NA |
| ASV_50 | 7.434398977 | Unassigned | Unassigned | NA |
| ASV_832 | 7.345743684 | Ascomycota | Archaeorhizomycetes | NA |
| ASV_955 | 7.343604087 | Ascomycota | Eurotiomycetes | Dung Saprotroph-Soil Saprotroph |
| ASV_96 | 7.270854998 | Ascomycota | Eurotiomycetes | Animal Pathogen-Fungal Parasite-Undefined Saprotroph |
| ASV_270 | 7.250071754 | Ascomycota | Sordariomycetes | NA |
| ASV_2658 | 7.242980179 | Ascomycota | Unassigned | NA |
| ASV_328 | 7.2305026 | Unassigned | Unassigned | NA |
| ASV_2097 | 7.1604269 | Ascomycota | Orbiliomycetes | Wood Saprotroph |
| ASV_553 | 7.043854678 | Unassigned | Unassigned | NA |
| ASV_2578 | 7.032333548 | Ascomycota | Unassigned | NA |
| ASV_813 | 7.003372747 | Ascomycota | Orbiliomycetes | Wood Saprotroph |
| ASV_104 | 7.000265581 | Ascomycota | Leotiomycetes | NA |
| ASV_306 | 6.984893687 | Ascomycota | Unassigned | NA |
| ASV_841 | 6.98232614 | Ascomycota | Eurotiomycetes | NA |
| ASV_389 | 6.958976797 | Ascomycota | Sordariomycetes | Undefined Saprotroph |
| ASV_109 | 6.946888356 | Ascomycota | Leotiomycetes | Undefined Saprotroph |
| ASV_437 | 6.934506901 | Ascomycota | Geoglossomycetes | Undefined Saprotroph |
| ASV_187 | 6.894432714 | Unassigned | Unassigned | NA |
| ASV_432 | 6.869056181 | Ascomycota | Dothideomycetes | NA |
| ASV_773 | 6.808613392 | Ascomycota | Eurotiomycetes | NA |
| ASV_114 | 6.78847465 | Ascomycota | Dothideomycetes | Wood Saprotroph |
| ASV_295 | 6.78227971 | Ascomycota | Unassigned | NA |
| ASV_213 | 6.777503324 | Ascomycota | Dothideomycetes | Fungal Parasite-Plant Pathogen-Plant Saprotroph |
| ASV_2367 | 6.749084823 | Ascomycota | Unassigned | NA |
| ASV_143 | 6.734735854 | Ascomycota | Dothideomycetes | Fungal Parasite-Plant Pathogen-Plant Saprotroph |
| ASV_240 | 6.702042583 | Ascomycota | Eurotiomycetes | NA |
| ASV_257 | 6.701441564 | Ascomycota | Unassigned | NA |
| ASV_241 | 6.699707017 | Ascomycota | Unassigned | NA |
| ASV_360 | 6.685851808 | Ascomycota | Leotiomycetes | NA |
| ASV_134 | 6.672965103 | Ascomycota | Archaeorhizomycetes | NA |
| ASV_2453 | 6.648024009 | Chlorophyta | Unassigned | NA |
| ASV_63 | 6.637634041 | Ascomycota | Eurotiomycetes | Animal Pathogen-Fungal Parasite-Undefined Saprotroph |
| ASV_893 | 6.624299592 | Ascomycota | Eurotiomycetes | NA |
| ASV_228 | 6.601776786 | Unassigned | Unassigned | NA |
| ASV_115 | 6.554834512 | Ascomycota | Sordariomycetes | Undefined Saprotroph |
| ASV_345 | 6.551556457 | Ascomycota | Sordariomycetes | NA |
| ASV_868 | 6.544688854 | Ascomycota | Unassigned | NA |
| ASV_406 | 6.537005736 | Ascomycota | Leotiomycetes | NA |
| ASV_2870 | 6.444440997 | Ascomycota | Orbiliomycetes | Wood Saprotroph |
| ASV_118 | 6.42730988 | Ascomycota | Eurotiomycetes | NA |
| ASV_330 | 6.411091171 | Ascomycota | Sordariomycetes | NA |
| ASV_321 | 6.35332717 | Unassigned | Unassigned | NA |
| ASV_52 | 6.346584998 | Ascomycota | Eurotiomycetes | NA |
| ASV_116 | 6.329581339 | Ascomycota | Sordariomycetes | Dung Saprotroph-Undefined Saprotroph |
| ASV_93 | 6.283037802 | Ascomycota | Unassigned | NA |
| ASV_2860 | 6.272274698 | Ascomycota | Eurotiomycetes | NA |
| ASV_3305 | 6.244883484 | Ascomycota | Eurotiomycetes | NA |
| ASV_3266 | 6.2084567 | Ascomycota | Eurotiomycetes | Animal Pathogen-Fungal Parasite-Undefined Saprotroph |
| ASV_160 | 6.192354422 | Ascomycota | Geoglossomycetes | NA |
| ASV_341 | 6.135971829 | Ascomycota | Unassigned | NA |
| ASV_205 | 6.131067102 | Ascomycota | Unassigned | NA |
| **ASV_371** | **6.130515318** | **Ascomycota** | **Pezizomycetes** | **Dung Saprotroph-Ectomycorrhizal-Soil Saprotroph-Wood Saprotroph** |
| ASV_333 | 6.118014743 | Ascomycota | Sordariomycetes | Undefined Saprotroph |
| ASV_794 | 6.09131018 | Ascomycota | Leotiomycetes | Undefined Saprotroph |
| **ASV_97** | **6.085539347** | **Ascomycota** | **Pezizomycetes** | **Dung Saprotroph-Ectomycorrhizal-Soil Saprotroph-Wood Saprotroph** |
| ASV_760 | 6.074309399 | Ascomycota | Leotiomycetes | NA |
| ASV_113 | 6.051210251 | Ascomycota | Sordariomycetes | Dung Saprotroph-Undefined Saprotroph |
| ASV_36 | 6.046828228 | Ascomycota | Eurotiomycetes | NA |
| ASV_363 | 6.025040039 | Ascomycota | Dothideomycetes | NA |
| ASV_3143 | 5.985570622 | Ascomycota | Geoglossomycetes | Undefined Saprotroph |
| **ASV_337** | **5.981978451** | **Ascomycota** | **Pezizomycetes** | **Dung Saprotroph-Ectomycorrhizal-Litter Saprotroph-Undefined Saprotroph** |
| ASV_163 | 5.957811418 | Ascomycota | Sordariomycetes | NA |
| **ASV_708** | **5.914652416** | **Ascomycota** | **Pezizomycetes** | **Dung Saprotroph-Ectomycorrhizal-Soil Saprotroph-Wood Saprotroph** |
| ASV_1041 | 5.828804142 | Ascomycota | Orbiliomycetes | Wood Saprotroph |

**Table S8** Proportions of the bacterial and fungal assembly processes during restoration.

|  | Restoration stage | undominated  processes | dispersal  limitation | homogenizing  dispersal | variable  selection | homogeneous  selection |
| --- | --- | --- | --- | --- | --- | --- |
| Bacteria | stage1 | 0.25% | 72.00% | 0.00% | 27.75% | 0.00% |
|  | stage2 | 0.26% | 41.32% | 0.00% | 58.42% | 0.00% |
|  | stage3 | 0.00% | 44.60% | 0.00% | 55.40% | 0.00% |
|  | stage4 | 0.00% | 57.37% | 0.00% | 42.63% | 0.00% |
| Fungi | stage1 | 44.50% | 3.50% | 6.50% | 45.50% | 0.00% |
|  | stage2 | 45.25% | 1.00% | 6.50% | 46.75% | 0.50% |
|  | stage3 | 52.00% | 1.50% | 5.00% | 41.50% | 0.00% |
|  | stage4 | 65.75% | 1.25% | 11.50% | 21.50% | 0.00% |

**Table S9** Adonis test of the functional genes on the basis of the Bray–Curtis distance.

|  | Df | Sum Of Sqs | R^2^ | F | Pr(>F) |
| --- | --- | --- | --- | --- | --- |
| Restoration year | 4 | 18.38 | 0.26 | 20.54 | 0.001 |
| Depth | 4 | 2.24 | 0.03 | 2.50 | 0.001 |
| Restoration year: Depth | 16 | 8.79 | 0.13 | 2.46 | 0.001 |
| Residual | 73 | 40.03 | 0.58 |  |  |
| Total | 97 | 69.44 | 1 |  |  |

**Table S10** Mantel test between soil physicochemical factors and the microbial community during restoration across soil depths on the basis of the Bray–Curtis distance.

|  |  | Topsoil | | Subsoil | |
| --- | --- | --- | --- | --- | --- |
|  |  | R^2^ | P | R^2^ | P |
| Bacteria | SOC | 0.385 | 0.0001 | 0.622 | 0.0001 |
|  | TN | 0.452 | 0.0001 | 0.646 | 0.0001 |
|  | TP | 0.052 | 0.1788 | 0.287 | 0.0001 |
|  | NH_4_^+^ | 0.026 | 0.3045 | 0.193 | 0.0024 |
|  | NO_3_^-^ | 0.254 | 0.0002 | 0.193 | 0.0006 |
|  | pH | 0.003 | 0.4525 | 0.056 | 0.1245 |
| Fungi | SOC | 0.157 | 0.0062 | 0.280 | 0.0001 |
|  | TN | 0.216 | 0.0016 | 0.240 | 0.0003 |
|  | TP | -0.023 | 0.6203 | 0.161 | 0.0018 |
|  | NH_4_^+^ | 0.197 | 0.0045 | 0.247 | 0.0003 |
|  | NO_3_^-^ | 0.235 | 0.0002 | 0.238 | 0.0004 |
|  | pH | -0.018 | 0.5964 | -0.042 | 0.8140 |

**Table S11** The 71 functional genes and primers used for qPCR.

| Gene_name | Classification | Functionality | Forward sequence | Reverse sequence |
| --- | --- | --- | --- | --- |
| abfA | C degradation | Hemicellulose hydrolysis | CGSTAYCCSGGCGGCAAYTT | TGCCASGGNCCGTCCATYTC |
| amyA | C degradation | Starch hydrolysis | YGGTTTTCGTCTTGACGCSG | MGGCTGMGTRTCATGRTTK |
| amyX | C degradation | Starch hydrolysis | TATAAYTGGGGMTATGAYCC | CCCATYAAATCAAAWCGRAA |
| apu | C degradation | Starch hydrolysis | ACVTGGATAGGYGAGCCYCA | CCRTCSGGGAAGTAGTTKCC |
| cdh | C degradation | Cellulose hydrolysis | ATWRYCTWCCGMRTHGCCMT | GTKAGSGGRTTBYKGRYCAT |
| cex | C degradation | Cellulose hydrolysis | YSTACGGSATGCACTGGMT | TANCGCAGRTAGTCVCCCAT |
| chiA | C degradation | Chitin hydrolysis | TSAAGAARTACGCSGACAACG | ASGTCATCAGRCCCTTSAG |
| exo-chi | C degradation | Chitin hydrolysis | GATTGGTSVCAATATGAYRG | STCCARCCACCRAYRCTRAA |
| glx | C degradation | Lignin hydrolysis | AACCAGTCGATCATCTACGA | RTGSACGAGCTCDGGCATGG |
| iso-plu | C degradation | Starch hydrolysis | GTCATYTACTTYGGNCC | CGNGCSACATCNGCCCA |
| lig | C degradation | Lignin hydrolysis | CCGCACACACTGTTGCTGC | CGAAGGATTGCCACTCGCA |
| manB | C degradation | Hemicellulose hydrolysis | ATGCGCGGBGTCAACCA | TCGTTGSCGATGTTGABGA |
| mnp | C degradation | Lignin hydrolysis | MACRCCSTTCGACTCSACC | ACGTCSGAGCAGTCRAYGA |
| naglu | C degradation | Cellulose hydrolysis | TVAAYTGGTAYCTGAAATAY | CCRTGYAGVGCCATCCAGTC |
| pgu | C degradation | Pectin hydrolysis | ANCATTGGTGGCCSTGGAA | TTRAYGGCRATRCARTCRTC |
| pox | C degradation | Lignin hydrolysis | ACYAGTATCCATTGGCACGGT | AGATGVGARTGATACCARAA |
| sga | C degradation | Starch hydrolysis | CGSAACTGGGAYTACCGS | TCCCACAGSCCSKCGTC |
| xylA | C degradation | Hemicellulose hydrolysis | TGGGGBGGTCGYGAAGG | ACTTTGGCRTCRAAGTT |
| accA | C fixation | C fixation | GAAGGCTAYCGCAARGC | CCTTCMGGSGARATMAC |
| aclB | C fixation | C fixation | TGGACMATGGTDGCYGGKGGT | ATAGTTKGGSCCACCTCTTC |
| acsA | C fixation | C fixation | GATACCTGGTGGCAGACCGA | TGATCACGTCGTCGACCCGG |
| acsB | C fixation | C fixation | CTYTGYCAGTCMTTYGCBCC | CCCATAAABCCYGGDGTYTG |
| acsE | C fixation | C fixation | TCATCGGCGAACGCATCAAC | AGRCCGGCTTCSATGGC |
| cdaR | C fixation | C fixation | CGARATGGTGGTGCTCAA | CARCGTRTTACGATGAATA |
| frdA | C fixation | C fixation | MTGCTGCACACSCTGTW | CCGGTSGGGTGRWACTG |
| korA | C fixation | C fixation | GCCGGCTACCCCATCACCCC | ATGATGGGATGGTCGCCATG |
| mct | C fixation | C fixation | TGGGCGCSGASGTSATMCG | TTGACSGTRTARTCSAYSGC |
| mcrA | C fixation | C fixation | GGTGGTGTMGGDTTCACMCARTA | CGTTCATBGCGTAGTTVGGRTAGT |
| pccA | C fixation | C fixation | GTGMTGATCAAGGCCWC | CGSGTGTTCATYTCSAGGAA |
| rbcL | C fixation | C fixation | AAGGAYGACGAGAACATC | TGCAGSATCATGTCRTT |
| smtA | C fixation | C fixation | TTTCTGGCCGGBTAYGCDGC | CGGTACGGHCCGGTYTGVCC |
| mmoX | Methane metabolism | Methane oxidation | ATGGAGGCGGTCAAGGACGA | CGCTTCATGCCCTTCCACAG |
| mxaF | Methane metabolism | Methane production | GCGGCACCAACTGGGGCTGGT | GGGCAGCATGAAGGGCTCCC |
| pqq-mdh | Methane metabolism | Methane production | TGTTCTATGTGCCGGCCAA | CTTCCACAGTTCCTTGCC |
| pmoA | Methane metabolism | Methane oxidation | GGNGACTGGGACTTCTGG | GAASGCNGAGAAGAASGC |
| amoA1 | N Cycling | Aerobic ammoxidation | STAATGGTCTGGCTTAGACG | GCGGCCATCCATCTGTATGT |
| amoA2 | N Cycling | Aerobic ammoxidation | GGGGTTTCTACTGGTGGT | CCCCTCKGSAAAGCCTTCTT |
| amoB | N Cycling | Aerobic ammoxidation | TGGTAYGACATKAWATGG | RCGSGGCARGAACATSGG |
| gdhA | N Cycling | Organic N mineralization | GCCATCGGYCCWTACAAGGG | ATGTCRCCNGCCGGAACGTC |
| hao | N Cycling | Nitrification | TGTCACATGGGTGTAGACCA | ACCTGGAACATACCCAT |
| hzo | N Cycling | Anaerobic ammonium oxidation | AAGACNTGYCAYTGGGGWAAA | GACATACCCATACTKGTRTANACNGT |
| hzsA | N Cycling | Anaerobic ammonium oxidation | WTYGGKTATCARTATGTAG | AAABGGYGAATCATARTGGC |
| hzsB | N Cycling | Anaerobic ammonium oxidation | ARGGHTGGGGHAGYTGGAAG | GTYCCHACRTCATGVGTCTG |
| napA | N Cycling | Denitrification | CTGGACIATGGGYTTIAACCA | CCTTCYTTYTCIACCCACAT |
| narG | N Cycling | Denitrification | TAYGTSGGGCAGGARAAACTG | CGTAGAAGAAGCTGGTGCTGT |
| nasA | N Cycling | Denitrification | CARCCNAAYGCNATGGG | ATNGTRTGCCAYTGRTC |
| nifH | N Cycling | Nfixation | AAAGGYGGWATCGGYAARTCCACCAC | TGSGCYTTGTCYTCRCGGATBGGCAT |
| nirK1 | N Cycling | Denitrification | GGMATGGTKCCSTGGCA | GCCTCGATCAGRTTRTGGTT |
| nirK2 | N Cycling | Denitrification | ATGGCGCCATCATGGTNYTNCC | TCGAAGGCCTCGATNARRTTRTG |
| nirK3 | N Cycling | Denitrification | TGCACATCGCCAACGGNATGTWYGG | GGCGCGGAAGATGSHRTGRTCNAC |
| nirS1 | N Cycling | Denitrification | GTSAACGTSAAGGARACSGG | GASTTCGGRTGSGTCTTGA |
| nirS2 | N Cycling | Denitrification | ATCGTCAACGTCAARGARACVGG | TTCGGGTGCGTCTTSABGAASAG |
| nirS3 | N Cycling | Denitrification | TGGAGAACGCCGGNCARGTNTGG | GATGATGTCCACGGCNACRTANGG |
| nosZ1 | N Cycling | Denitrification | CGCRACGGCAASAAGGTSMSSGT | CAKRTGCAKSGCRTGGCAGAA |
| nosZ2 | N Cycling | Denitrification | CGYTGTTCMTCGACAGCCAG | CGSACCTTSTTGCCSTYGCG |
| nxrA | N Cycling | Nitrification | CAGACCGACGTGTGCGAAAG | TCCACAAGGAACGGAAGGTC |
| ureC | N Cycling | Ammonification | AAGMTSCACGAGGACTGGGG | AGRTGGTGGCASACCATSAGCAT |
| bpp | P Cycling | Organic P mineralization | GACGCAGCCGAYGAYCCNGCNITNTGG | CAGGSCGCANRTCIACRTTRTT |
| cphy | P Cycling | Organic P mineralization | GTGGACCTRCGRMARGARWCICA | GTCCGACCATTGCCTGCYTCRCART GRAMRTGIADCCA |
| gcd | P Cycling | Inorganic P solubilization | ATCGCGTTCGGGCCGGACG | ATSAGRTTSAGCTCGTCCCA |
| phnK | P Cycling | Organic P mineralization | CATCGTCGGCGAATCCGG | TGCTGCATGCCGCCGGAAAA |
| phoD | P Cycling | Organic P mineralization | CAGTGGGACGACCACGAGGT | GAGGCCGATCGGCATGTCG |
| phoX | P Cycling | Organic P mineralization | GARGAGAACWTCCACGGYTA | GATCTCGATGATRTGRCCRAAG |
| ppk | P Cycling | Inorganic P biosynthesis | GACCCGAABGTRCTBGCSAT | TTATAATTNCCSGTNCCNA |
| ppx | P Cycling | Inorganic P hydrolysis | TGCATCTGGCGGACGGCCT | AGATCCGCCGCCAATATCA |
| pqqC | P Cycling | Inorganic P solubilization | AACCGCTTCTACTACCAG | GCGAACAGCTCGGTCAG |
| apsA | S Cycling | S reduction | GGGYCTKTCCGCYATCAAYAC | ATCATGATCTGCCAGCGGCCGGA |
| dsrA | S Cycling | S reduction | ACSCACTGGAAGCACG | GGTGGAGCCGTGCATGTT |
| dsrB | S Cycling | S reduction | CAACATCGTYCAYACCCAGGG | GTGTAGCAGTTACCGCA |
| soxY | S Cycling | S oxidation | ATCGATGACAACCCCGTGCC | AGCTGGTCCATCTGCATGCCG |
| yedZ | S Cycling | S oxidation | CTGCTGATCACGCTGGCCAT | GCGATGCAGCTTCTTCCAGCG |
